# Convalescent Plasma Therapy for COVID-19: A Systematic Review and Meta-Analysis on Randomized Controlled Trials

**DOI:** 10.3390/v15030765

**Published:** 2023-03-16

**Authors:** Charalampos Filippatos, Ioannis Ntanasis-Stathopoulos, Kalliopi Sekeri, Anastasios Ntanasis-Stathopoulos, Maria Gavriatopoulou, Theodora Psaltopoulou, George Dounias, Theodoros N. Sergentanis, Evangelos Terpos

**Affiliations:** 1 Department of Clinical Therapeutics, School of Medicine, National and Kapodistrian University of Athens, 11528 Athens, Greece; 2 Department of Public Health Policy, School of Public Health, University of West Attica, 11521 Athens, Greece

**Keywords:** COVID-19, convalescent plasma, meta-analysis, randomized controlled trials, mortality, intensive care unit

## Abstract

**Background:** While passive immunotherapy has been considered beneficial for patients with severe respiratory viral infections, the treatment of COVID-19 cases with convalescent plasma produced mixed results. Thus, there is a lack of certainty and consensus regarding its effectiveness. This meta-analysis aims to assess the role of convalescent plasma treatment on the clinical outcomes of COVID-19 patients enrolled in randomized controlled trials (RCTs). **Methods:** A systematic search was conducted in the PubMed database (end-of-search: 29 December 2022) for RCTs on convalescent plasma therapy compared to supportive care\standard of care. Pooled relative risk (RR) and 95% confidence intervals were calculated with random-effects models. Subgroup and meta-regression analyses were also performed, in order to address heterogeneity and examine any potential association between the factors that varied, and the outcomes reported. The present meta-analysis was performed following the Preferred Reporting Items for Systematic Reviews and Meta-Analyses (PRISMA) guidelines. **Results:** A total of 34 studies were included in the meta-analysis. Per overall analysis, convalescent plasma treatment was not associated with lower 28-day mortality [RR = 0.98, 95% CI (0.91, 1.06)] or improved 28-day secondary outcomes, such as hospital discharge [RR = 1.00, 95% CI (0.97, 1.03)], ICU-related or score-related outcomes, with effect estimates of RR = 1.00, 95% CI (0.98, 1.05) and RR = 1.06, 95% CI (0.95, 1.17), respectively. However, COVID-19 outpatients treated with convalescent plasma had a 26% less risk of requiring hospital care, when compared to those treated with the standard of care [RR = 0.74, 95% CI (0.56, 0.99)]. Regarding subgroup analyses, COVID-19 patients treated with convalescent plasma had an 8% lower risk of ICU-related disease progression when compared to those treated with the standard of care (with or without placebo or standard plasma infusions) [RR = 0.92, 95% CI (0.85, 0.99)] based on reported outcomes from RCTs carried out in Europe. Finally, convalescent plasma treatment was not associated with improved survival or clinical outcomes in the 14-day subgroup analyses. **Conclusions:** Outpatients with COVID-19 treated with convalescent plasma had a statistically significantly lower risk of requiring hospital care when compared to those treated with placebo or the standard of care. However, convalescent plasma treatment was not statistically associated with prolonged survival or improved clinical outcomes when compared to placebo or the standard of care, per overall analysis in hospitalized populations. This hints at potential benefits, when used early, to prevent progression to severe disease. Finally, convalescent plasma was significantly associated with better ICU-related outcomes in trials carried out in Europe. Well-designed prospective studies could clarify its potential benefit for specific subpopulations in the post-pandemic era.

## 1. Introduction

At the end of 2019, a surge of pneumonia cases in Wuhan, a city in the Hubei Province of China, led to the identification of a novel coronavirus as the cause. Its rapid spread resulted in an epidemic throughout China, followed by an increasing number of cases around the world. On 11 March 2020, the World Health Organization declared the novel coronavirus outbreak a pandemic. The disease associated with it was designated as COVID-19, which stands for coronavirus disease 2019, and the virus that caused it was designated as Severe Acute Respiratory Syndrome Coronavirus 2 (SARS-CoV-2) [1].

As of the end of 2022, COVID-19 disease management involves mostly supportive care, symptomatic treatment and prevention by vaccination. There have been only a few drugs or treatments proven to be effective specifically against the virus and the illness it causes, such as nirmatrelvir\ritonavir for high-risk patients.

Convalescent plasma, having been used to treat viral outbreaks of novel infectious diseases affecting the respiratory system in the past [2,3], was an early candidate [4,5]. The idea behind it is to transfuse blood plasma from a person who has recovered from a specific illness to someone who currently has the same illness in order to provide passive immunity and boost their fight against the pathogen, since such plasma contains antibodies to it [6,7].

With the potential for convalescent plasma to be beneficial, there was an urgency for clinical trials. The FDA provided emergency use authorization for its use and the WHO reinforced clinical trials to continue enrolment. Later updates included revisions on the matter, such as the focus and authorization being shifted to immunosuppressed patients or outpatients (FDA) or severe and high-risk patients in general (WHO) [8]. Moreover, the most up-to-date emergency authorization letter by the FDA states that convalescent plasma units used should be “high-titer”, based on studies showing the superiority of high-titer convalescent plasma in terms of preventing severe COVID-19-related outcomes [9].

Because studies and reviews yielded conflicting results, there has been a persistent lack of certainty and consensus regarding its efficacy [10,11]. Therefore, we conducted a meta-analysis, focusing strictly on RCTs, to assess the effect of convalescent plasma treatment on the clinical outcomes of patients with COVID-19.

## 2. Materials and Methods

### 2.1. Search Strategy and Eligibility of Studies

The present meta-analysis was performed following the Preferred Reporting Items for Systematic Reviews and Meta-Analyses (PRISMA) guidelines [12]. The study protocol was discussed and agreed upon in advance by all authors.

A systematic search was conducted in the PubMed database, using the following algorithm: 

(COVID-19 OR SARS-CoV-2 OR “novel coronavirus”) AND (convalescent OR convalescence) AND (plasma OR serum).

Eligible articles included randomized clinical trials on convalescent plasma treatment vs. supportive care or standard of care controls, with or without placebo. Case-control, cohort and cross-sectional studies, case series and case reports, reviews, in vitro and animal studies were not included in this meta-analysis. The selection of studies was conducted initially by two co-authors (CF and ANS) by independent work and any disagreements were resolved following consultation with a senior author (INS or TNS) and team consensus.

### 2.2. Data Abstraction and Effect Estimates

The data abstraction encompassed: general information (first author’s name, publication year, PubMed and CT database ID), study characteristics (time period, follow-up period, geographic region, multicenter status, control type, participant numbers, percentage of males, age), intervention characteristics (time to intervention from symptom onset and total CP dose) and outcomes (mortality and clinical outcomes with reported effect estimates or fourfolds with plain data, adjustment details).

If one of the above was not found in the main article, the Supplementary Material was thoroughly screened. There was no shortage of required data for the purposes of the meta-analysis. Data were independently extracted, analyzed and recorded in separate data extraction sheets by two authors (CF and KS). The finalized data form was reached after consultation with a senior author (TNS) and team consensus.

Extracted effect estimates included relative risks alongside their 95% Cis (per outcome) or any other form that could be mathematically transformed or translated to relative risk. Mortality was extracted as a primary outcome for our work and hospitalization, and hospital discharge, ICU-related outcomes and score-related outcomes were secondary outcomes.

As far as score-related outcomes are concerned, all of them were based on or using variations of the 9-point WHO score for COVID-19. This is defined as: 0: no clinical or virological evidence of infection; 1: ambulatory, no activity limitation; 2: ambulatory, activity limitation; 3: hospitalized, no oxygen therapy; 4: hospitalized, oxygen mask or nasal prongs; 5: hospitalized, noninvasive mechanical ventilation (NIMV) or high-flow nasal cannula (HFNC); 6: hospitalized, intubation and invasive mechanical ventilation (IMV); 7: hospitalized, IMV + additional support such as pressors or extracardiac membranous oxygenation (ECMO); 8: death.

Finally, a titer subgroup analysis was carried out, between studies that fulfilled the latest EUA/FDA cut-offs for high-titer plasma units versus the rest. This is defined as a neutralizing antibody titer of ≥250 in the Broad Institute’s neutralizing antibody assay or an S/C cutoff of ≥12 in the Ortho VITROS IgG assay.

In case the aforementioned information was not available, crude effect estimates and 95% CIs were calculated by means of fourfolds from plain data extracted from the articles.

### 2.3. Statistical Analyses

Statistical analyses included pooling of studies as well as post hoc meta-regression. Random-effects models were appropriately used to calculate the pooled effect estimates (relative risks). The convalescent plasma treatment arms were compared to the control arms. Between-study heterogeneity was assessed by Q-test and I^2^ estimations. Subgroup analyses were performed based on adjustment, multicenter status, blinding status and the geographic region of each study.

The post hoc meta-regression analysis was performed for subgroups with a total of 10 or more data entries for the variables to be analyzed. The aim was to assess whether gender, age, time from symptom onset to intervention or total convalescent plasma dose modified the association between convalescent plasma transfusion and each reported outcome.

All statistical analyses were performed using STATA/SE version 13 (Stata Corp, College Station, TX, USA).

### 2.4. Assessment of Study Quality and Risk of Bias

All records included randomized clinical trials, either blinded or open label. Risk was assessed with the implementation of the RoB:2 algorithm by Cochrane to our analysis tools [11]. Specifically, two authors (KS and ANS) carried out the assessment procedure independently, and upon inspection of the results by a third author (CF), consensus was met.

Publication bias was evaluated in the analyses that included 10 or more study arms [13]. For this purpose, Egger’s statistical test (statistical significance *p* < 0.1) [14,15] was implemented as well as the funnel plot inspection. The evaluation of publication bias was performed using STATA/SE version 13 (Stata Corp, College Station, TX, USA).

## 3. Results

### 3.1. Description of Eligible Studies

**Figure 1:**
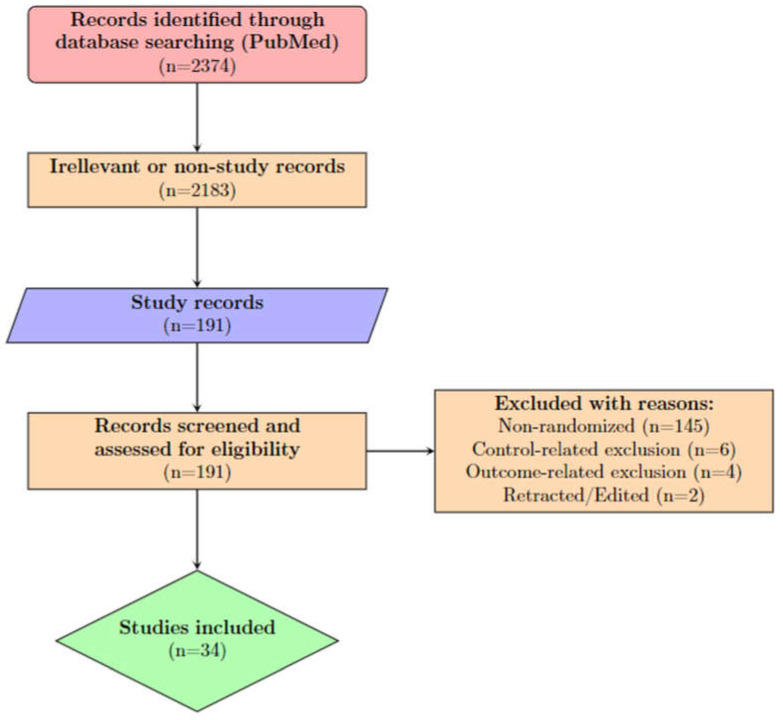
Study selection flowchart.

A total of 2374 records were identified from PubMed using the search algorithm (Section 2.1) and were assessed for eligibility. The flowchart (Figure 1) portrays the successive steps in the selection of eligible studies.

For the 28-day main cohort, 34 randomized controlled trials were included [16,17,18,19,20,21,22,23,24,25,26,27,28,29,30,31,32,33,34,35,36,37,38,39,40,41,42,43,44,45,46,47,48,49]. For the 14-day secondary cohort, 10 articles on randomized controlled trials provided the necessary data. All studies had convalescent plasma therapy arms vs. standard of care or supportive care arms, with some including standard plasma, non-convalescent plasma or fresh-frozen plasma to the control arms.

From the 28-day cohort studies, all of them reported mortality figures, except one (Alemany, 2022) [17]. Regarding secondary clinical outcomes, hospital discharge was reported on nine records, ICU-related outcomes were reported on twenty-one records, hospitalization was a reported outcome in six studies and score-related outcomes (WHO score for COVID-19) in six studies.

Table 1 and Table 2 present the characteristics of the included studies regarding study design, patient and disease characteristics and interventions.

### 3.2. Meta-Analysis

#### 3.2.1. 28-Day Results

**Figure 2:**
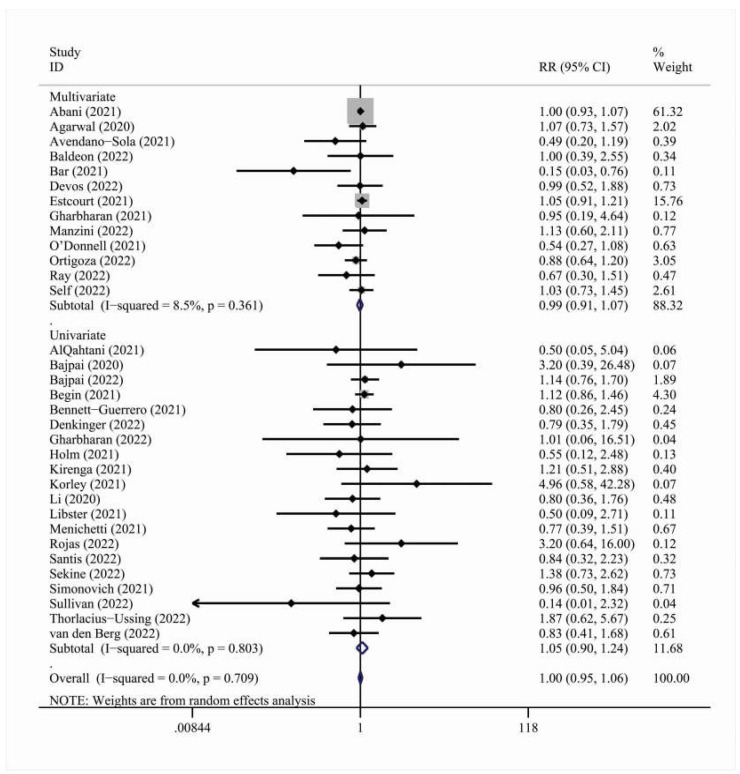
Forest plot describing the association between convalescent plasma treatment and 28-day mortality. Apart from the overall analysis, the subanalysis on adjustment type is presented.

In total, 34 studies were included in the overall meta-analysis for the 28-day cohort [13,14,15,16,17,18,19,20,21,22,23,24,25,26,27,28,29,30,31,32,33,34,35,36,37,38,39,40,41,42,43,44,45,46]. The effect outcome for 28-day mortality was not statistically significant [RR = 1.00, 95% C.I. (0.95, 1.06)] (Figure 2). There were no statistically significant results in the adjustment, multicenter status, blinding status and geographic region subgroup analyses (Table 3).

A meta-analysis for the secondary clinical outcomes showed no statistically significant association between convalescent plasma therapy and hospital discharge [RR = 0.99, 95% C.I. (0.96, 1.03)] or score-related outcomes [RR = 1.06, 95% C.I. (0.97, 1.16)] (Table 4 and Table 5). The ICU-related outcomes analysis yielded no statistically significant overall result [RR = 0.98, 95% C.I. (0.93, 1.02)] as well (Table 6). 

Furthermore, a subgroup analysis was conducted according to the levels of anti-SARS-CoV-2 antibodies in the CP. Studies were grouped as “high-titer” or “non-high-titer” as per the latest EUA/FDA guideline cut-offs. The subgroup analysis for the titer level did not reveal any statistically significant associations (Supplementary Figures S5, S10, S14, S20 and S25). 

**Figure 3:**
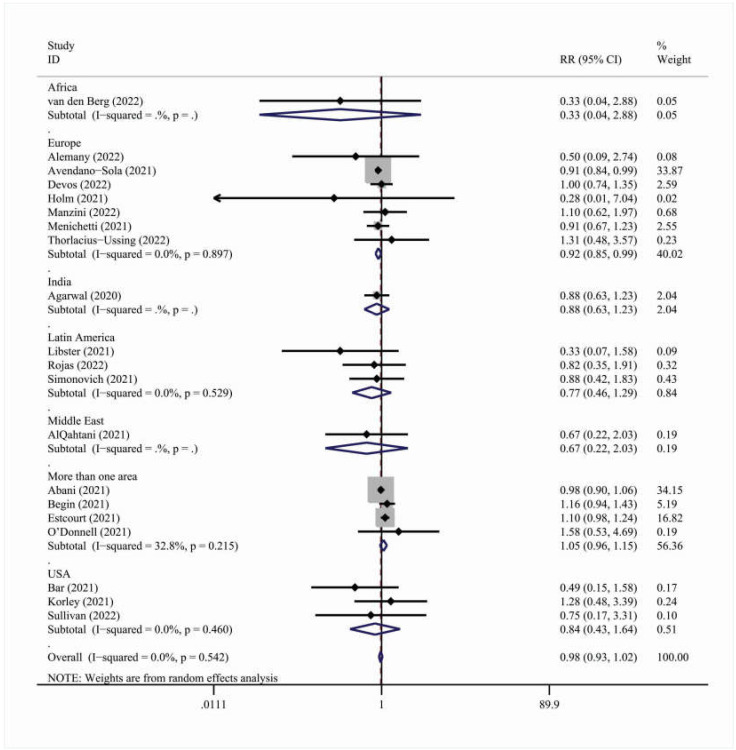
Forest plot describing the association between convalescent plasma treatment and 28-day ICU-related outcomes. Apart from the overall analysis, the subanalysis per geographic region is presented.

However, when analyzing by geographic region, studies carried out in Europe [RR = 0.92, 95% C.I. (0.85, 0.99)] showed a statistically significant association between convalescent plasma therapy and ICU-related outcomes (Table 6 and Figure 3).

**Figure 4:**
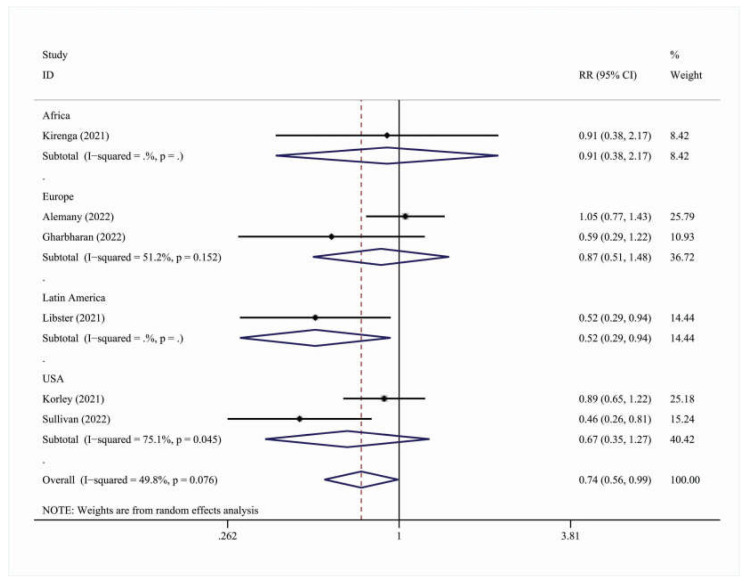
Forest plot describing the association between convalescent plasma treatment and 28-day hospitalization outcomes. Apart from the overall analysis, the subanalysis on geographic region is presented.

The subanalysis on hospitalization outcomes [RR = 0.74, 95% C.I. (0.56, 0.99)] was also statistically significant, showing that outpatients treated with convalescent plasma had a 26% less risk of needing hospital care than those treated with the standard of care (Table 7, Figure 4).

#### 3.2.2. 14-Day Results

**Figure 5:**
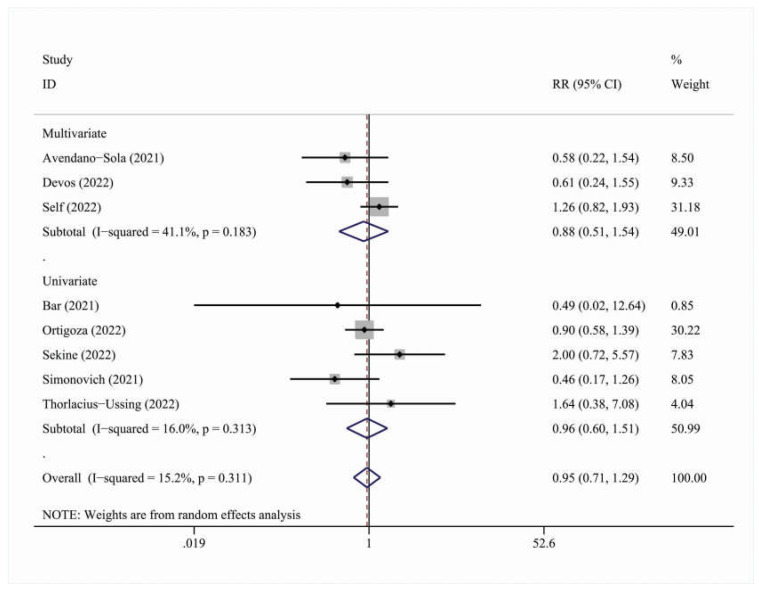
Forest plot describing the association between convalescent plasma treatment and 14-day mortality. Apart from the overall analysis, the subanalysis on adjustment type is presented.

In total, 10 studies were included in the overall meta-analysis for the 14-day cohort. The effect outcome for 14-day mortality was not statistically significant [RR = 0.98, 95% C.I. (0.91, 1.06)] (Figure 5 and Table 8). There were no statistically significant results in the adjustment, multicenter status, blinding status or geographic region subgroups (Table 8).

A meta-analysis for the secondary clinical outcomes showed no statistically significant association between convalescent plasma therapy and hospital discharge [RR = 0.96, 95% C.I. (0.89, 1.03)] (Table 9). A subgroup analysis for the titer level was not statistically significant as well (Supplementary Figures S30 and S35). 

### 3.3. Meta-Regression Analysis

The post hoc meta-regression aimed to assess whether gender, age, time from symptom onset to intervention or total cp dose modified the association between convalescent plasma treatment and each reported outcome. This analysis yielded no statistically significant associations (Table 10 and Table 11). It was carried out only for the 28-day analysis cohort and specifically only for the overall mortality, hospital discharge and ICU-related outcomes, as other categories had less than 10 study arms.

### 3.4. Quality Assessment and Risk of Bias

All included studies were randomized control trials, blinded or open label. For the evaluation of quality and risk of bias of each one, the RoB:2 tool by Cochrane was used [13]. Table 12 presents the risk of bias assessment for the included studies. 

In total, 23/34 studies (67.7%) were assessed as having a low risk of bias, 10/34 studies (29.4%) raised some concerns and only one was deemed to have a high risk of bias [40]. More specifically:

- Six studies (17.7%) raised some concerns on their randomization process, mostly due to lack of information on allocation concealment;
- Five studies (14.7%) raised some concerns on whether there were deviations from the intended interventions;
- Only one study raised concerns on potential selection of the reported result;
- One study had a high risk of bias due to vital randomization process concerns.

### 3.5. Publication Bias

A publication bias assessment was performed on outcomes reported in 10 or more studies with the use of Egger’s test [14,15]. These were the 28-day mortality and 28-day ICU-related outcomes.

For the 28-day mortality analysis, for a total of 33 studies, the *p*-value for the bias coefficient generated by Egger’s regression test for small-study effects was *p* = 0.247 (Supplemental Figure S35). For the 28-day ICU-related outcomes, for a total of 20 studies, the aforementioned *p*-value was *p* = 0.337 (Supplemental Figure S36). In both cases, this means that there were no small-study effects, and thus no publication bias.

## 4. Discussion

The present meta-analysis, comprising data from 34 individual randomized controlled trials, found no statistically significant association between convalescent plasma treatment and 28-day or 14-day mortality, hospital discharge, hospitalization, ICU-related or score-related outcomes. When analyzing by subgroups, though, the European cohort for the ICU-related outcomes yielded a statistically significant result [RR = 0.92, 95% CI (0.85, 0.99)], showing that convalescent plasma treatment was beneficial in protecting patients from ICU-related disease progression. Specifically, patients treated with convalescent plasma had an 8% less risk of presenting an ICU-related outcome (such as the need for ventilation treatment, intubation, ECMO), when compared to those treated with the standard of care or supportive care (with or without placebo/standard plasma infusion). While this result is interesting and significant, it can largely be attributed to the contribution of the weight of the Avendaño-Solá (2021) study [17]. Moreover, convalescent plasma was found to be beneficial in protecting outpatients from hospitalization. After analyzing hospitalization outcomes, a statistically significant result [RR = 0.74, 95% C.I. (0.56, 0.99)] was yielded, meaning that outpatients treated with convalescent plasma had a 26% lower risk of needing to be hospitalized than those treated with the standard of care.

Carrying out a subgroup analysis for titer levels (high-titer vs. non-high titer) was challenging, as each study used different antibody measurements and cut-off levels for high-titer labeling. Moreover, achieving in-study heterogeneity among the titers of the plasma units administered was also significant. These led to a statistically nonsignificant and mostly inconclusive result. There was a scarcity of outcome data regarding secondary clinical outcomes, such as hospital discharge (9/34 studies), hospitalization (6/34 studies) and score-related outcomes (6/34 studies). The plasma titer between studies varied and so did COVID-19 disease severity at randomization and study size. Serostatus at the time of treatment was not possible to assess and analyze, as only a percentage of studies provided robust and uniform data for it. Furthermore, records were extracted solely from the PubMed database.

In addition, the RECOVERY trial (Abani, 2021) has raised some concerns during our risk of bias assessment and is worth mentioning, as its weight skewed the results. This is due to the fact that it failed to completely adhere to its design, as 9% of the patients did not receive the allocated intervention (plasma infusion). While this raises questions about the robustness of the results, the aforementioned population percentage was excluded from the comparison analysis between the convalescent plasma group and the control group.

Despite the aforementioned notable limitations, the present work possesses a plethora of important strengths. Overall heterogeneity was low and not significant both in the 28-day (I^2^ = 0.0%, *p* = 0.709) and 14-day (I^2^ = 15.2%, *p* = 0.311) cohorts. In the statistically significant ICU-related European subgroup, heterogeneity was also low and not significant (I^2^ = 0.0%, *p* = 0.897). Overall heterogeneity was 49.8% for the hospitalization outcomes subanalysis, but it was marginally not statistically significant (*p* = 0.076). While region, sex, age, time from symptom onset to intervention and total convalescent plasma dose can be considerable sources of heterogeneity, subgroup analyses and meta-regression showed no statistically significant association between them and treatment effectiveness. The extensive abstraction and analysis of separate and discrete clinical outcomes and thorough risk of bias assessment are also parts of this study’s strengths. Contrary to other meta-analyses [50,51,52], our work focuses strictly on randomized controlled trials, thus lying in the highest part of the hierarchy of evidence pyramid. 

Moreover, screening was extensive and detailed, pairing information from each trial article and its official registry page. This led to avoiding errors such as misclassifying [50,51,52] the article record by Rasheed et al. [53] as an RCT, when it was a control-matched cohort study. Furthermore, thorough auditing led to excluding two trials, which were retracted/edited as far as their patient allocation method was concerned.

When comparing our work to others, the results for overall mortality (a commonly reported primary outcome) are similar. Axfors et al. conducted a systematic review and meta-analysis on 33 published and unpublished trial papers and showed a non-statistically significant association as well [54]. Other published meta-analyses were comprised of considerably fewer studies, such as the study by Piscova et al. [50] with five trials and six cohorts, the analysis by Snow et al. [51] with seventeen trials and the study by Janiaud et al. including ten trials [55]. The meta-analysis by Kloypan et al. [52] showed a statistically significant association between convalescent plasma therapy and overall mortality but it was subject to notable limitations. The primary outcome of all-cause mortality at any given time point included nonrandomized trials and observational studies, whereas the Rasheed trial was misclassified. Another difference lies in our secondary outcomes analysis, where the aforementioned systematic reviews and meta-analyses failed to yield statistically significant results. This can be attributed to the big pool of studies (and thus variety and data available), outcome assessment and categorization and extensive subgroup analyses.

Finally, subgroup analyses on immunocompromised patients were not feasible due to the scarcity of available data from randomized studies in the field. A recently published randomized controlled trial by Dekinger et al. [49] evaluated the role of convalescent plasma in a subgroup of 56 patients with hematological or solid cancer and severe COVID-19. The administration of convalescent plasma significantly improved survival and reduced the time to clinical improvement. Patients with cancer under active treatment present attenuated humoral responses to COVID-19 vaccination, and thus they are at high risk for severe SARS-CoV-2 infection [56,57,58,59]. Other trials on vulnerable populations for severe COVID-19-related outcomes showed signs of benefits with [35] or without statistically significant results [30,33]. A recent systematic review and meta-analysis including trials, cohort studies, case series and case reports found that convalescent plasma therapy was associated with a mortality benefit in patients who were immunocompromised and were diagnosed with COVID-19 [60,61].

## 5. Conclusions

Convalescent plasma treatment was not associated with a statistically significant reduced risk of overall 28-day or 14-mortality or any other clinical outcome. It was associated, though, with a statistically significant beneficial effect on 28-day ICU-related outcomes in the European study cohort and 28-day hospitalization. The aforementioned evidence hints against the use of convalescent plasma for the treatment of COVID-19 in the general population, but it highlights potential clinical benefits when studying subpopulations (e.g., European ICU cohorts, outpatients). As such, further study on specific subpopulations and outcomes could establish consensus on determining the clinical benefits of convalescent plasma therapy.

## Figures and Tables

**Table 1:** General characteristics of the included studies.

Author and Year	Setting	Geographic Region	Multicenter	Blinded	Control
Abani (2021)	Hospitalized	More than one area	Yes	No	SoC
Agarwal (2020)	Hospitalized	India	Yes	No	SoC
Alemany (2022)	Outpatients	Europe	Yes	Yes	Placebo
AlQahtani (2021)	Hospitalized	Middle East	No	No	SoC
Avendaño-Solá (2021)	Hospitalized	Europe	Yes	No	SoC
Bajpai (2020)	Hospitalized	India	No	No	SoC
Bajpai (2022)	Hospitalized	India	Yes	No	SoC
Baldeón (2022)	Hospitalized	Latin America	Yes	Yes	Placebo
Bar (2021)	Hospitalized	USA	No	No	SoC
Bégin (2021)	Hospitalized	More than one area	Yes	No	SoC
Bennett-Guerrero (2021)	Hospitalized	USA	No	Yes	Placebo
Dekinger (2022)	Hospitalized	Europe	Yes	No	SoC
Devos (2022)	Hospitalized	Europe	Yes	No	SoC
Estcourt (2021)	Hospitalized	More than one area	Yes	No	SoC
Gharbharan (2021)	Hospitalized	Europe	Yes	No	SoC
Gharbharan (2022)	Outpatients	Europe	Yes	Yes	Placebo
Holm (2021)	Hospitalized	Europe	No	No	SoC
Kirenga (2021)	Mixed	Africa	No	No	SoC
Korley (2021)	Outpatients	USA	Yes	Yes	SoC
Li (2020)	Hospitalized	East Asia	Yes	No	SoC
Libster (2021)	Outpatients	Latin America	Yes	Yes	Placebo
Manzini (2022)	Hospitalized	Europe	No	Yes	SoC
Menichetti (2021)	Hospitalized	Europe	Yes	No	SoC
O’Donnell (2021)	Hospitalized	More than one area	Yes	Yes	Placebo
Ortigoza (2022)	Hospitalized	USA	Yes	Yes	Placebo
Ray (2022)	Hospitalized	India	No	No	SoC
Rojas (2022)	Hospitalized	Latin America	Yes	Yes	SoC
Santis (2022)	Hospitalized	Latin America	Yes	No	SoC
Sekine (2022)	Hospitalized	Latin America	No	Yes	SoC
Self (2022)	Hospitalized	USA	Yes	Yes	Placebo
Simonovich (2021)	Hospitalized	Latin America	No	Yes	Placebo
Sullivan (2022)	Outpatients	USA	Yes	Yes	Placebo
Thorlacius-Ussing (2022)	Hospitalized	Europe	Yes	No	Placebo
van de Berg (2022)	Hospitalized	Africa	No	Yes	Placebo

SoC: standard of care; placebo control includes SoC.

**Table 2:** Intervention characteristics of the included studies.

Author and Year	CP (n)	Control (n)	Male %	Age (μ ± σ)	Time from Symptom Onset to Intervention (μ ± σ)	CP Dose (mL)
Abani (2021)	5795	5763	64%	63.50 ± 14.70	9.00 ± 4.45	550
Agarwal (2020)	235	229	76%	51.13 ± 19.53	8.35 ± 3.73	400
Alemany (2022)	188	188	54%	56.70 ± 7.44	4.40 ± 1.40	275
AlQahtani (2021)	20	20	80%	51.65 ± 19.45	10.00	400
Avendaño-Solá (2021)	179	171	65%	63.00 ± 15.30	5.65 ± 2.23	275
Bajpai (2020)	14	15	73%	48.20 ± 9.80	3.00	500
Bajpai (2022)	200	200	67%	55.52 ± 1.17	-	500
Baldeón (2022)	63	95	68%	74.34 ± 18.39	10.60 ± 4.90	-
Bar (2021)	40	39	46%	-	7.71 ± 4.53	-
Bégin (2021)	625	313	59%	67.50 ± 15.60	7.90 ± 3.70	500
Bennett-Guerrero (2021)	59	15	60%	65.70 ± 23.50	11.12 ± 9.12	480
Denkinger (2022)	68	66	68%	68.50 ± 11.30	7.00 ± 4.50	575
Devos (2022)	320	163	69%	62.00 ± 14.00	7.00 ± 4.46	450
Estcourt (2021)	1078	909	68%	60.77 ± 18.38	-	550
Gharbharan (2021)	43	43	72%	64.40 ± 13.45	10.35 ± 6.72	300
Gharbharan (2022)	207	209	78%	60.00 ± 7.44	5.00 ± 1.49	400
Holm (2021)	17	14	61%	69.95 ± 40.64	7.00 ± 3.23	675
Kirenga (2021)	69	67	71%	50.18 ± 17.61	6.30 ± 3.00	-
Korley (2021)	257	254	46%	51.90 ± 16.35	3.65 ± 2.24	250
Li (2020)	52	51	58%	70.00 ± 12.03	29.65 ± 14.29	-
Libster (2021)	80	80	38%	77.20 ± 8.60	1.65 ± 0.58	250
Manzini (2022)	60	60	72%	65.48 ± 11.96	8.35 ± 5.23	600
Menichetti (2021)	232	241	64%	64.00 ± 14.87	7.21 ± 2.98	400
O’Donnell (2021)	150	73	66%	60.30 ± 17.91	10 ± 4.49	-
Ortigoza (2022)	468	473	59%	62.65 ± 15.59	6.65 ± 3.71	250
Ray (2022)	40	40	71%	-	4.20 ± 2.21	400
Rojas (2022)	46	45	70%	51.76 ± 18.68	10.65 ± 2.96	500
Santis (2022)	36	71	72%	56.00 ± 16.16	9.00 ± 1.50	1800
Sekine (2022)	80	80	41%	58.74 ± 14.96	10.00 ± 3.00	300
Self (2022)	487	473	57%	59.65 ± 15.59	7.65 ± 3.72	300
Simonovich (2021)	228	105	67%	62.00 ± 14.89	7.65 ± 3.72	500
Sullivan (2022)	592	589	57%	43.35 ± 23.12	5.65 ± 2.23	250
Thorlacius-Ussing (2022)	98	46	72%	65.00 ± 14.98	10.65 ± 3.76	600
van de Berg (2022)	52	51	41%	77.20 ± 8.60	8.65 ± 3.76	250

Missing values were not reported either in the article or in the supplementary material. CP dose is the total convalescent plasma transfused.

**Table 3:** Results of the meta-analyses examining the association between convalescent plasma therapy and mortality (28-day); subgroup analyses by adjustment, multicenter status, blinding status and geographic region are presented.

	*n*	*RR*	*Heterogeneity I^2^, p*
Overall analysis	33	0.98 (0.91, 1.06)	0.0%, 0.709
**Subgroups by adjustment**			
Multivariate	13	0.99 (0.91, 1.07)	8.5%, 0.361
Univariate	20	1.05 (0.95, 1.06)	0.0%, 0.803
**Subgroups by multicenter status**			
Multicenter	22	1.00 (0.95, 1.06)	0.0%, 0.703
Single-center	11	0.95 (0.74, 1.24)	0.0, 0.451
**Subgroups by blinding status**			
Blinded	15	0.97 (0.82, 1.15)	0.0%, 0.524
Open label	18	1.01 (0.95, 1.07)	0.0%, 0.667
**Subgroups by geographic region**			
Africa	2	0.96 (0.56, 1.67)	0.0%, 0.509
East Asia	1	0.80 (0.36, 1.76)	Not calculable
Europe	9	0.89 (0.67, 1.19)	0.0%, 0.778
India	4	1.06 (0.82, 1.38)	0.0%, 0.493
Latin America	6	1.10 (0.77, 1.57)	0.0%, 0.623
Middle East	1	0.50 (0.05, 5.04)	Not calculable
USA	6	0.85 (0.55, 1.31)	47.1%, 0.092
More than one area	4	1.02 (0.93, 1.12)	26.8%, 0.251

Highlighted rows denote statistically significant associations.

**Table 4:** Results of the meta-analyses examining the association between convalescent plasma therapy and hospital discharge (28-day); subgroup analyses by adjustment, multicenter status, blinding status and geographic region are presented.

	*n*	*RR*	*Heterogeneity I^2^, p*
Overall analysis	9	0.99 (0.96, 1.03)	0.0%, 0.955
**Subgroups by adjustment**			
Multivariate	2	0.98 (0.94, 1.03)	0.0%, 0.455
Univariate	7	1.01 (0.96, 1.05)	0.0%, 0.949
**Subgroups by multicenter status**			
Multicenter	7	1.00 (0.96, 1.03)	0.0%, 0.859
Single-center	2	0.95 (0.74, 1.24)	0.0, 0.451
**Subgroups by blinding status**			
Blinded	15	0.97 (0.82, 1.15)	0.0%, 0.524
Open label	18	0.98 (0.87, 1.11)	0.0%, 1.000
**Subgroups by geographic region**			
Africa	1	0.98 (0.72, 1.34)	Not calculable
Europe	1	1.06 (0.87, 1.30)	Not calculable
India	1	1.03 (0.88, 1.21)	Not calculable
Latin America	2	1.00 (0.92, 1.09)	0.0%, 0.652
USA	2	0.99 (0.94, 1.06)	0.0%, 0.529
More than one area	2	1.00 (0.92 1.08)	21.5%, 0.259

Highlighted rows denote statistically significant associations.

**Table 5:** Results of the meta-analyses examining the association between convalescent plasma therapy and score-related outcomes (28-day); subgroup analyses by adjustment, multicenter status, blinding status and geographic region are presented.

	*n*	*RR*	*Heterogeneity I^2^, p*
Overall analysis	7	1.06 (0.97, 1.16)	17.2%, 0.299
**Subgroups by adjustment**			
Multivariate	2	1.25 (0.87, 1.78)	61.0%, 0.110
Univariate	5	1.01 (0.94, 1.09)	0.0%, 0.601
**Subgroups by multicenter status**			
Multicenter	4	1.15 (1.02, 1.29)	0.0%, 0.451
Single-center	3	0.99 (0.91, 1.07)	0.0%, 0.854
**Subgroups by blinding status**			
Blinded	4	1.12 (0.98, 1.27)	0.0%, 0.394
Open label	3	1.03 (0.92, 1.17)	21.5%, 0.280
**Subgroups by geographic region**			
Africa	2	0.98 (0.90, 1.07)	0.0%, 0.749
East Asia	1	1.20 (0.82, 1.75)	Not calculable
Europe	1	1.16 (0.91, 1.47)	Not calculable
Latin America	1	1.60 (1.03, 2.49)	Not calculable
USA	1	1.33 (0.37, 4.77)	Not calculable
More than one area	1	1.09 (0.93, 1.27)	Not calculable

Highlighted rows denote statistically significant associations.

**Table 6:** Results of the meta-analyses examining the association between convalescent plasma therapy and ICU-related outcomes (28-day); subgroup analyses by adjustment, multicenter status, blinding status and geographic region are presented.

	*n*	*RR*	*Heterogeneity I^2^, p*
Overall analysis	20	0.98 (0.93, 1.02)	0.0%, 0.542
**Subgroups by adjustment**			
Multivariate	3	1.00 (0.86, 1.15)	70.7%, 0.033
Univariate	17	0.98 (0.93, 1.02)	0.0%, 0.542
**Subgroups by multicenter status**			
Multicenter	14	0.98 (0.93, 1.04)	8.0%, 0.365
Single-center	6	0.84 (0.57, 1.24)	0.0%, 0.703
**Subgroups by blinding status**			
Blinded	10	0.96 (0.71, 1.31)	8.0%, 0.807
Open label	10	0.99 (0.92, 1.06)	27.4%, 0.192
**Subgroups by geographic region**			
Africa	1	0.33 (0.04, 2.88)	Not calculable
Europe	7	0.92 (0.85, 0.99)	0.0%, 0.897
India	1	0.88 (0.63, 1.23)	Not calculable
Latin America	3	0.77 (0.46, 1.29)	0.0%, 0.529
Middle East	1	0.67 (0.22, 2.03)	Not calculable
USA	3	0.84 (0.43, 1.64)	0.0%, 0.460
More than one area	4	1.05 (0.96, 1.15)	32.8%, 0.215
**Subgroups by ICU-related outcome**			
ICU admission	4	0.97 (0.74, 1.26)	0.0%, 0.501
IMV or ECMO or death	1	1.10 (0.98, 1.24)	Not calculable
Intubation or death	1	1.16 (0.94, 1.43)	Not calculable
IMV	1	0.33 (0.04, 2.88)	Not calculable
Invasive ventilatory support	1	0.88 (0.42, 1.83)	Not calculable
MV	1	0.50 (0.09, 2.74)	Not calculable
MV or ICU admission	1	0.75 (0.17, 3.31)	Not calculable
MV or death	1	1.10 (0.62, 1.97)	Not calculable
MV or ECMO	1	0.49 (0.15, 1.58)	Not calculable
NIV or high flow O2 or IMV or ECMO or death	1	0.91 (0.84, 0.99)	Not calculable
PaO2/FiO2 of <150 mm Hg or death	1	0.91 (0.67, 1.23)	Not calculable
Ventilation treatment	6	0.98 (0.90, 1.06)	0.0%, 0.784

Highlighted rows denote statistically significant associations.

**Table 7:** Subanalysis on hospitalization (28-day).

	*n*	*RR*	*Heterogeneity I^2^, p*
Overall analysis	6	0.74 (0.56, 0.99)	49.8%, 0.076
**Subgroups by adjustment**			
Multivariate	0	-	-
Univariate	6	0.74 (0.56, 0.99)	49.8%, 0.076
**Subgroups by multicenter status**			
Multicenter	5	0.72 (0.52, 1.00)	59.6%, 0.042
Single-center	1	0.91 (0.38, 2.17)	Not calculable
**Subgroups by blinding status**			
Blinded	5	0.72 (0.52, 1.00)	59.6%, 0.042
Open label	1	0.91 (0.38, 2.17)	Not calculable
**Subgroups by geographic region**			
Africa	1	0.91 (0.38, 2.17)	Not calculable
Europe	2	0.87 (0.51, 1.48)	51.2%, 0.152
Latin America	1	0.52 (0.29, 0.94)	Not calculable
USA	2	0.67 (0.35, 1.27)	75.1%, 0.045

Table 7 Results of the meta-analyses examining the association between convalescent plasma therapy and hospitalization outcomes (28-day); subgroup analyses by adjustment, multicenter status, blinding status and geographic region are presented. Highlighted rows denote statistically significant associations.

**Table 8:** Results of the meta-analyses examining the association between convalescent plasma therapy and overall mortality (14-day); subgroup analyses by adjustment, multicenter status, blinding status and geographic region are presented.

	*n*	*RR*	*Heterogeneity I^2^, p*
Overall analysis	8	0.95 (0.71, 1.29)	15.2%, 0.311
**Subgroups by adjustment**			
Multivariate	3	0.88 (0.51, 1.54)	41.1%, 0.183
Univariate	5	0.96 (0.60, 1.51)	6.0%, 0.313
**Subgroups by multicenter status**			
Multicenter	5	0.98 (0.75, 1.30)	1.4%, 0.398
Single-center	3	0.89 (0.28, 2.83)	51.9%, 0.125
**Subgroups by blinding status**			
Blinded	4	1.03 (0.68, 1.57)	43.1%, 0.153
Open label	4	0.70 (0.38, 1.28)	0.0%, 0.666
**Subgroups by geographic region**			
Europe	3	0.71 (0.31, 1.31)	0.0%, 0.467
Latin America	2	0.96 (0.23, 4.04)	75.1%, 0.045
USA	3	1.06 (0.78, 1.44)	0.0%, 0.500

Highlighted rows denote statistically significant associations.

**Table 9:** Results of the meta-analyses examining the association between convalescent plasma therapy and hospital discharge (14-day); subgroup analyses by adjustment, multicenter status, blinding status and geographic region are presented.

	*n*	*RR*	*Heterogeneity I^2^, p*
Overall analysis	4	0.96 (0.89, 1.03)	0.0%, 0.995
**Subgroups by adjustment**			
Multivariate	-	-	-
Univariate	4	0.96 (0.89, 1.03)	0.0%, 0.995
**Subgroups by multicenter status**			
Multicenter	2	0.96 (0.88, 1.04)	0.0%, 0.795
Single-center	2	0.96 (0.81, 1.14)	0.0%, 0.964
**Subgroups by blinding status**			
Blinded	3	0.96 (0.88, 1.04)	0.0%, 0.999
Open label	1	0.93 (0.75, 1.16)	Not calculable
**Subgroups by geographic region**			
Europe	1	0.93 (0.75, 1.16)	Not calculable
Latin America	2	0.96 (0.81, 1.14)	0.0%, 0.964
USA	1	0.96 (0.87, 1.05)	Not calculable

Highlighted rows denote statistically significant associations.

**Table 10:** Meta-regression on mortality (28-day). Results of meta-regression analysis examining the role of potential modifiers in the association between convalescent plasma treatment and 28-day mortality.

Variables	Increment	*n*	Exponentiated Coefficient	*p*
Male%	10% increase	33	1.06 (0.93, 1.21)	0.368
Mean age	10 y increase	31	0.92 (0.76, 1.12)	0.405
Time from symptom onset to intervention	1 day more	31	1.00 (0.97, 1.04)	0.945
Total CP dose	100 mL more	27	1.01 (0.96, 1.07)	0.691

**Table 11:** Results of meta-regression analysis examining the role of potential modifiers in the association between convalescent plasma treatment and 28-day ICU-related outcomes.

Variables	Increment	*n*	Exponentiated Coefficient	*p*
Male%	10% increase	20	1.02 (0.85, 1.23)	0.789
Mean age	10 y increase	19	1.07 (0.86, 1.35)	0.514
Time from symptom onset to intervention	1 day more	19	1.03 (0.99, 1.06)	0.157
Total CP dose	100 mL more	18	1.05 (1.00, 1.11)	0.064

**Table 12:** Risk of bias assessment based on the RoB:2 algorithm.

	Randomization Process	Deviations from Intended Interventions	Missing Outcome Data	Measurement of the Outcome	Selection of the Reported Result	Overall
Abani (2021)	Low risk	Some concerns	Low risk	Low risk	Low risk	Some concerns
Agarwal (2020)	Low risk	Low risk	Low risk	Low risk	Low risk	Low risk
Alemany (2022)	Low risk	Low risk	Low risk	Low risk	Low risk	Low risk
AlQahtani (2021)	Low risk	Low risk	Low risk	Low risk	Low risk	Low risk
Avendaño-Solá (2021)	Low risk	Low risk	Low risk	Low risk	Low risk	Low risk
Bajpai (2020)	Some concerns	Low risk	Low risk	Low risk	Low risk	Some concerns
Bajpai (2022)	Low risk	Low risk	Low risk	Low risk	Low risk	Low risk
Baldeón (2022)	Low risk	Low risk	Low risk	Low risk	Low risk	Low risk
Bar (2021)	Some concerns	Low risk	Low risk	Low risk	Low risk	Some concerns
Bégin (2021)	Low risk	Low risk	Low risk	Low risk	Low risk	Low risk
Bennett-Guerrero (2021)	Low risk	Low risk	Low risk	Low risk	Low risk	Low risk
Denkinger (2022)	Some concerns	Low risk	Low risk	Low risk	Low risk	Some concerns
Devos (2022)	Low risk	Low risk	Low risk	Low risk	Low risk	Low risk
Estcourt (2021)	Low risk	Low risk	Low risk	Low risk	Low risk	Low risk
Gharbharan (2021)	Low risk	Some concerns	Low risk	Low risk	Low risk	Some concerns
Gharbharan (2022)	Low risk	Low risk	Low risk	Low risk	Low risk	Low risk
Holm (2021)	Some concerns	Some concerns	Low risk	Low risk	Low risk	Some concerns
Kirenga (2021)	Low risk	Low risk	Low risk	Low risk	Low risk	Low risk
Korley (2021)	Low risk	Low risk	Low risk	Low risk	Low risk	Low risk
Li (2020)	Some concerns	Low risk	Low risk	Low risk	Low risk	Some concerns
Libster (2021)	Low risk	Low risk	Low risk	Low risk	Low risk	Low risk
Manzini (2022)	Some concerns	Low risk	Low risk	Low risk	Low risk	Some concerns
Menichetti (2021)	Low risk	Low risk	Low risk	Low risk	Low risk	Low risk
O’Donnell (2021)	Low risk	Low risk	Low risk	Low risk	Some concerns	Some concerns
Ortigoza (2022)	Low risk	Low risk	Low risk	Low risk	Low risk	Low risk
Ray (2022)	High risk	Some concerns	Low risk	Low risk	Low risk	High risk
Rojas (2022)	Low risk	Low risk	Low risk	Low risk	Low risk	Low risk
Santis (2022)	Low risk	Some concerns	Some concerns	Low risk	Low risk	Some concerns
Sekine (2022)	Low risk	Low risk	Low risk	Low risk	Low risk	Low risk
Self (2022)	Low risk	Low risk	Low risk	Low risk	Low risk	Low risk
Simonovich (2021)	Low risk	Low risk	Low risk	Low risk	Low risk	Low risk
Sullivan (2021)	Low risk	Low risk	Low risk	Low risk	Low risk	Low risk
Thorlacius-Ussing (2022)	Low risk	Low risk	Low risk	Low risk	Low risk	Low risk
van de Berg (2022)	Low risk	Low risk	Low risk	Low risk	Low risk	Low risk

## Data Availability

Data available upon reasonable request from the corresponding author.

## Supplementary Materials

The following supporting information can be downloaded at: https://www.mdpi.com/article/10.3390/v15030765/s1. Figure S1. 28-day mortality, by adjustment; Figure S2. 28-day mortality, by multicenter status; Figure S3. 28-day mortality, by blinding status; Figure S4. 28-day mortality, by geographic region; Figure S5. 28-day mortality, by titer; Figure S6. 28-day hospitalization, by adjustment; Figure S7. 28-day hospitalization, by multicenter status; Figure S8. 28-day hospitalization, by blinding status; Figure S9. 28-day hospitalization, by geographic region; Figure S10. 28-day hospitalization, by titer; Figure S11. 28-day hospital discharge, by adjustment; Figure S12. 28-day hospital discharge, by multicenter status; Figure S13. 28-day hospital discharge, by blinding status; Figure S14. 28-day hospital discharge, by geographic region; Figure S15. 28-day hospital discharge, by titer; Figure S16. 28-day ICU-related outcomes, by adjustment; Figure S17. 28-day ICU-related outcomes, by multicenter status; Figure S18. 28-day ICU-related outcomes, by adjustment; Figure S19. 28-day ICU-related outcomes, by geographic region; Figure S20. 28-day ICU-related outcomes, by ICU status; Figure S21. 28-day ICU-related outcomes, by titer; Figure S22. 28-day score-related outcomes, by adjustment; Figure S23. 28-day score-related outcomes, by multicenter status; Figure S24. 28-day score-related outcomes, by blinding status; Figure S25. 28-day score-related outcomes, by geographic region; Figure S26. 28-day score-related outcomes, by titer; Figure S27. 14-day mortality, by adjustment; Figure S28. 14-day mortality, by multicenter status; Figure S29. 14-day mortality, by blinding status; Figure S30. 14-day mortality, by geographic region; Figure S31. 14-day mortality, by titer; Figure S32. 14-day hospital discharge, by adjustment; Figure S33. 14-day hospital discharge, by multicenter status; Figure S34. 14-day hospital discharge, by blinding status; Figure S35. 14-day hospital discharge, by multicenter status; Figure S36. 14-day hospital discharge, by titer; Figure S37. Funnel plot, portraying publication bias for 28-day mortality; Figure S38. Funnel plot, portraying publication bias for 28-day ICU-related outcomes
