# Supplementary material for: Convalescent Plasma Therapy for COVID-19: A Systematic Review and Meta-Analysis on Randomized Controlled Trials
Source: Viruses. 2023 Mar 16;15(3):765. doi: 10.3390/v15030765 (PMC10054551; doi:10.3390/v15030765)

Convalescent Plasma therapy for COVID-19:

A systematic review and meta-analysis on Randomized Controlled Trials

Supplementary material

Supplementary Figure S1. 28-day mortality, by adjustment

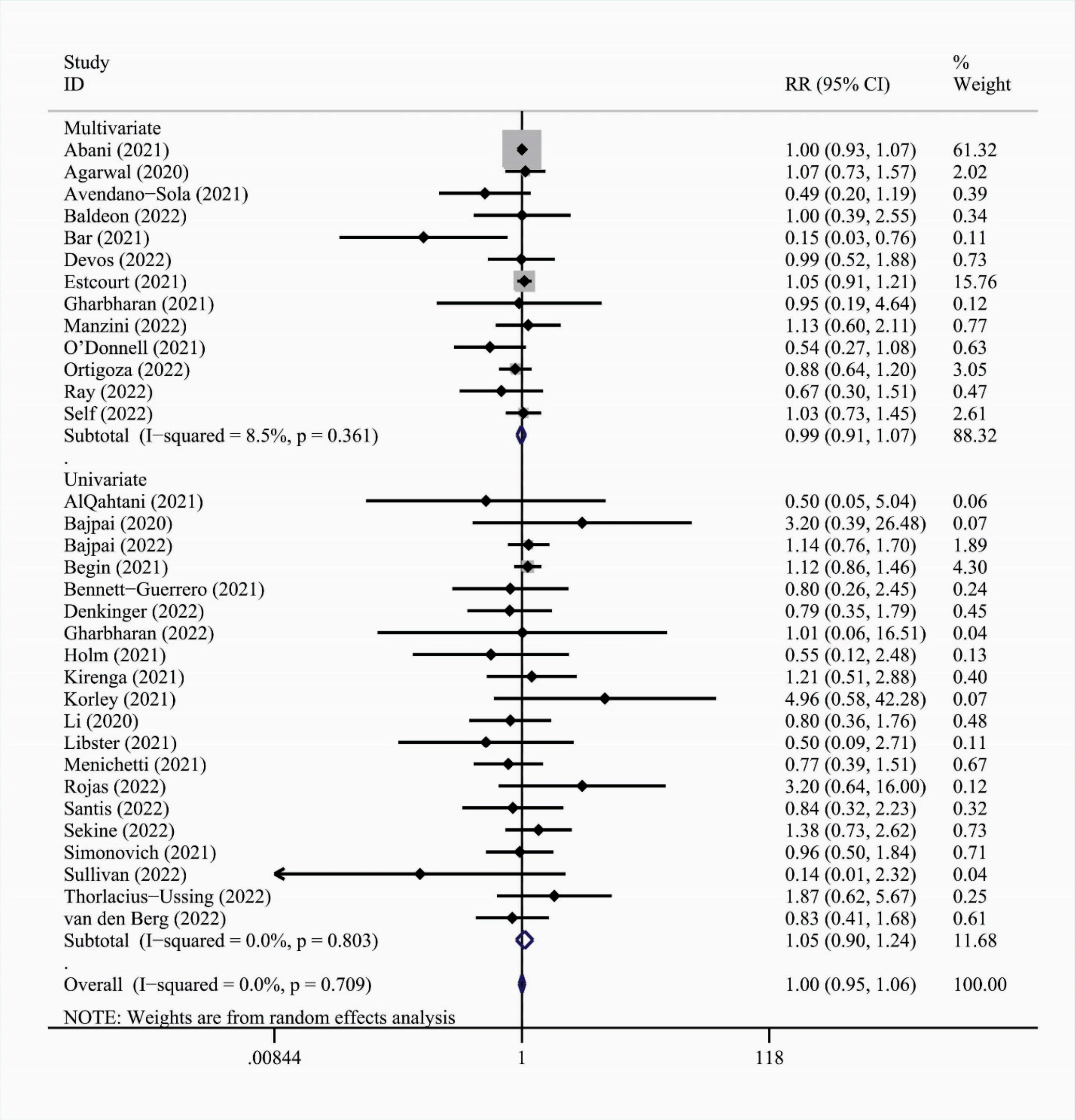

Figure S1. Forest plot describing the association between convalescent plasma treatment and 28-day mortality. Apart from the overall analysis, the sub-analysis on adjustment type is presented.

Supplementary Figure S2. 28-day mortality, by multicenter status

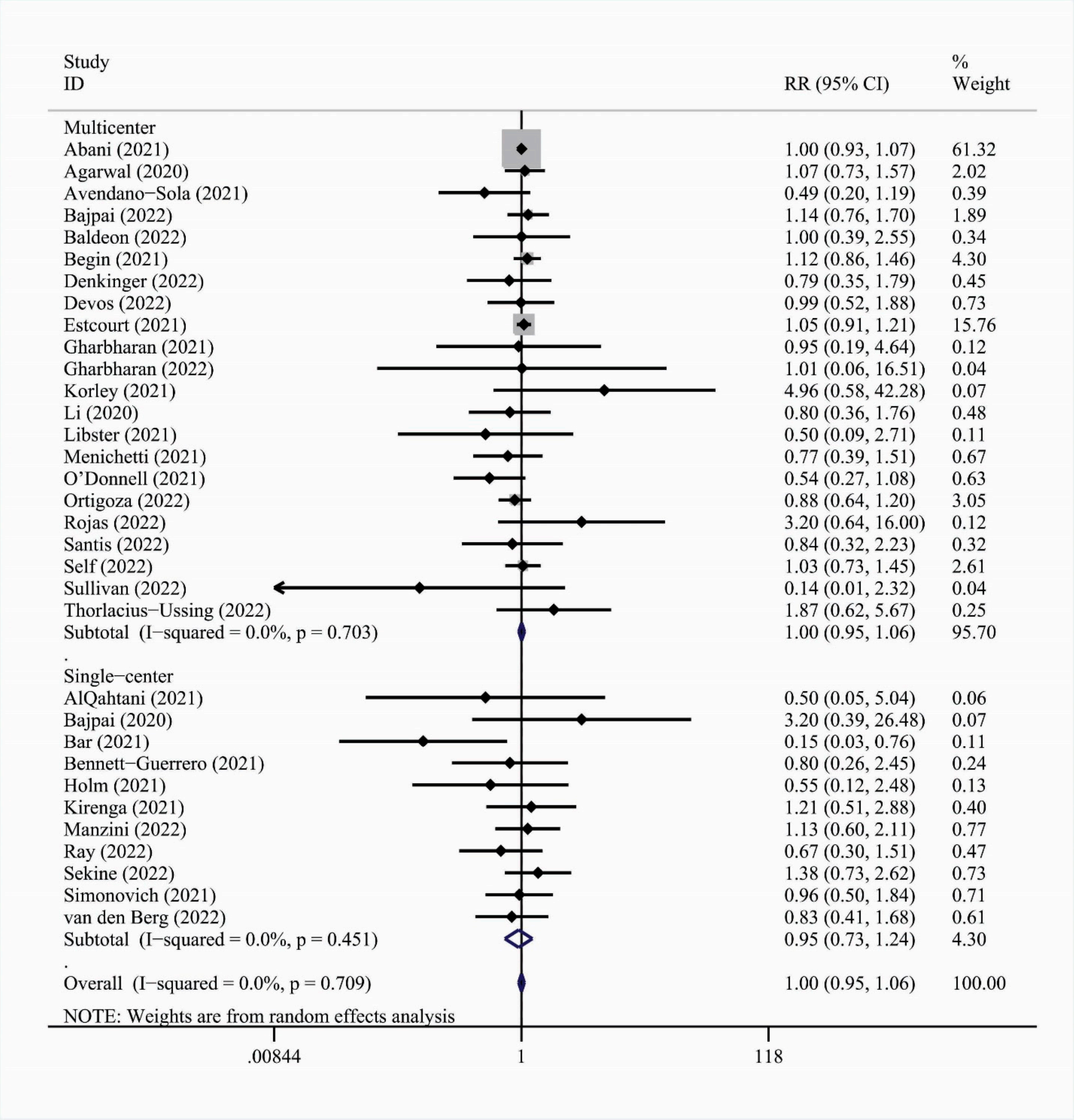

Figure S2. Forest plot describing the association between convalescent plasma treatment and 28-day mortality. Apart from the overall analysis, the sub-analysis on multicenter status is presented.

Supplementary Figure S3. 28-day mortality, by blinding status

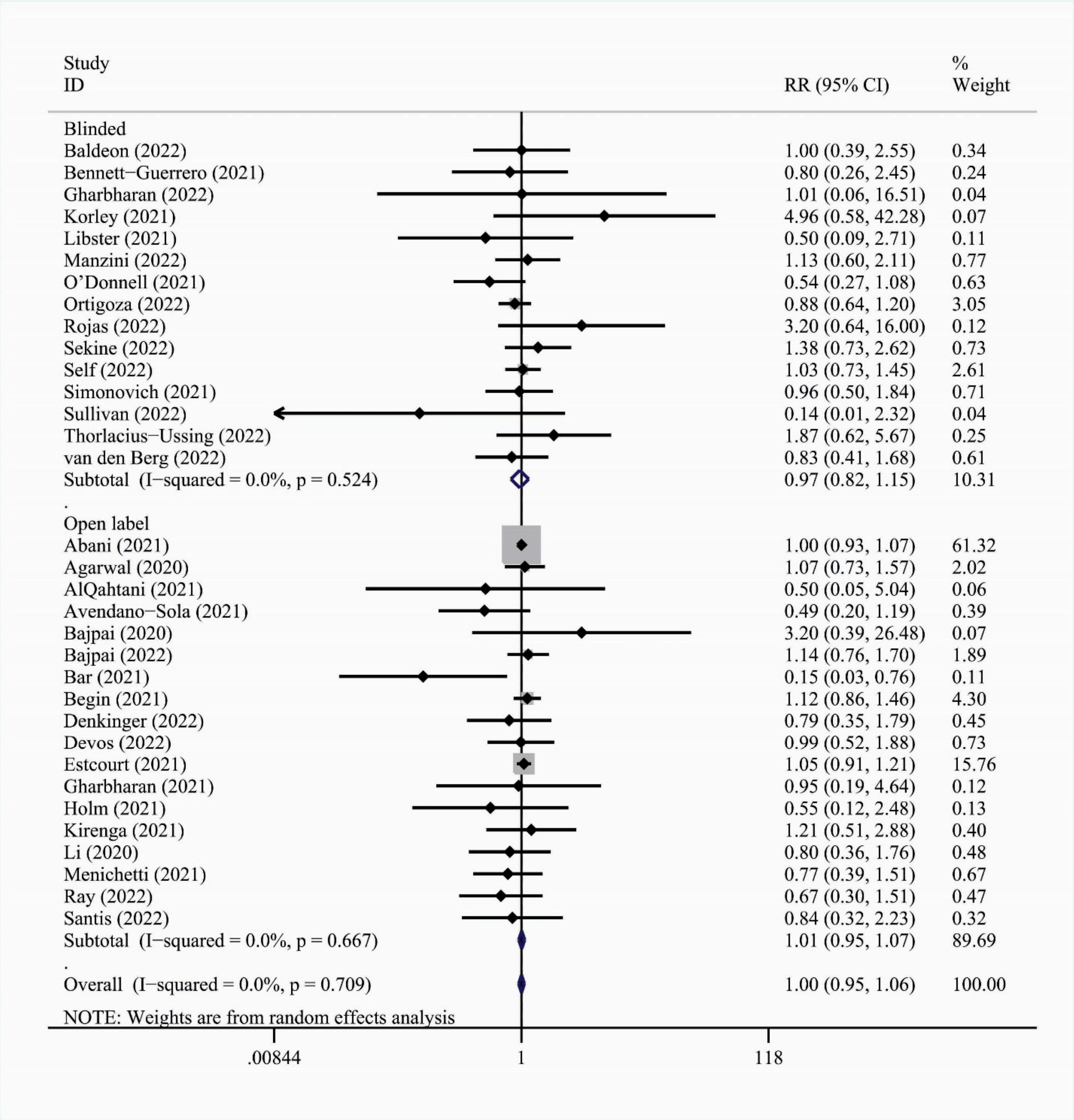

Figure S3. Forest plot describing the association between convalescent plasma treatment and 28-day mortality. Apart from the overall analysis, the sub-analysis on blinding status is presented.

Supplementary Figure S4. 28-day mortality, by geographic region

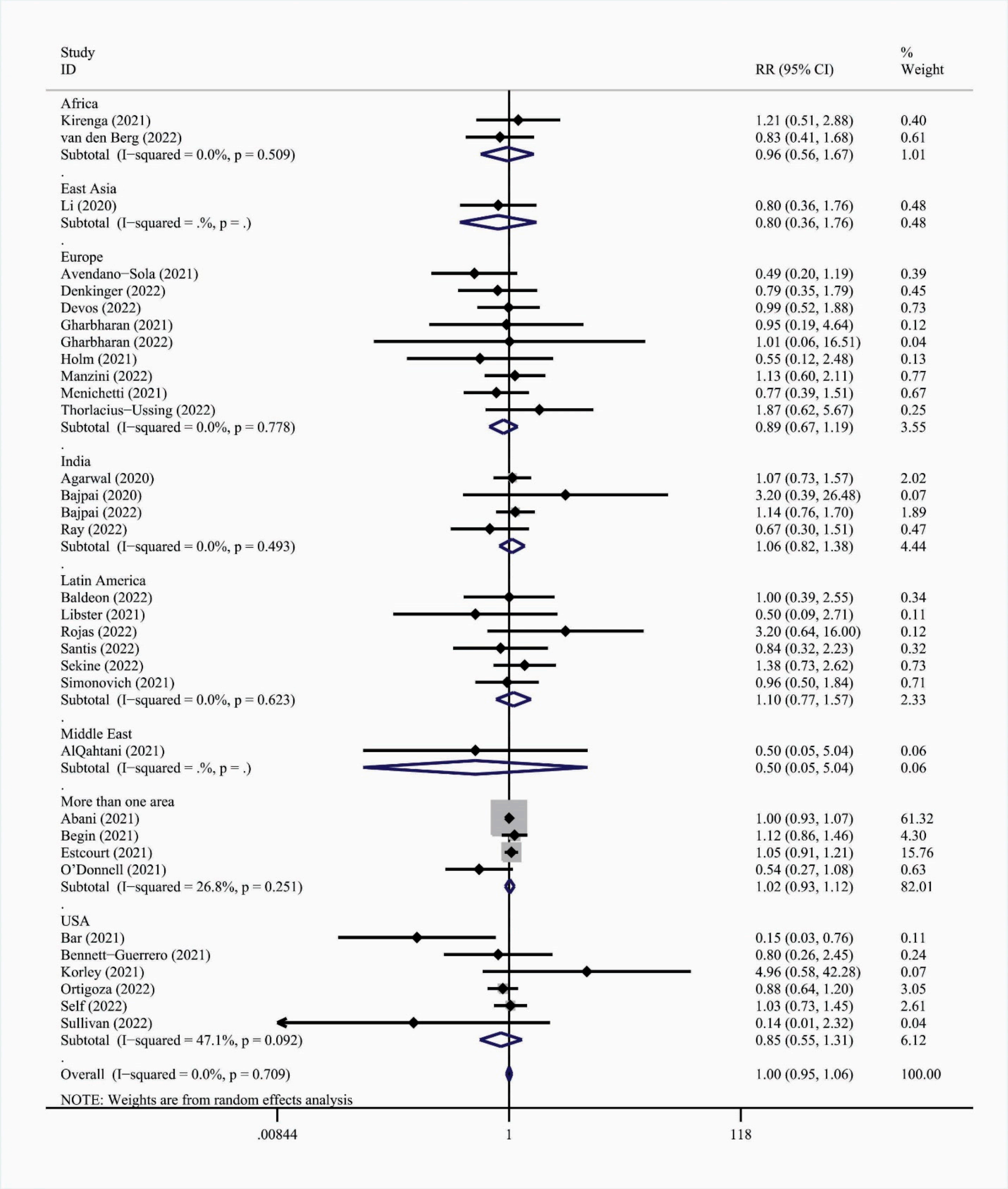

Figure S4. Forest plot describing the association between convalescent plasma treatment and 28-day mortality. Apart from the overall analysis, the sub-analysis by geographic region is presented.

Supplementary Figure S5. 28-day mortality, by titer

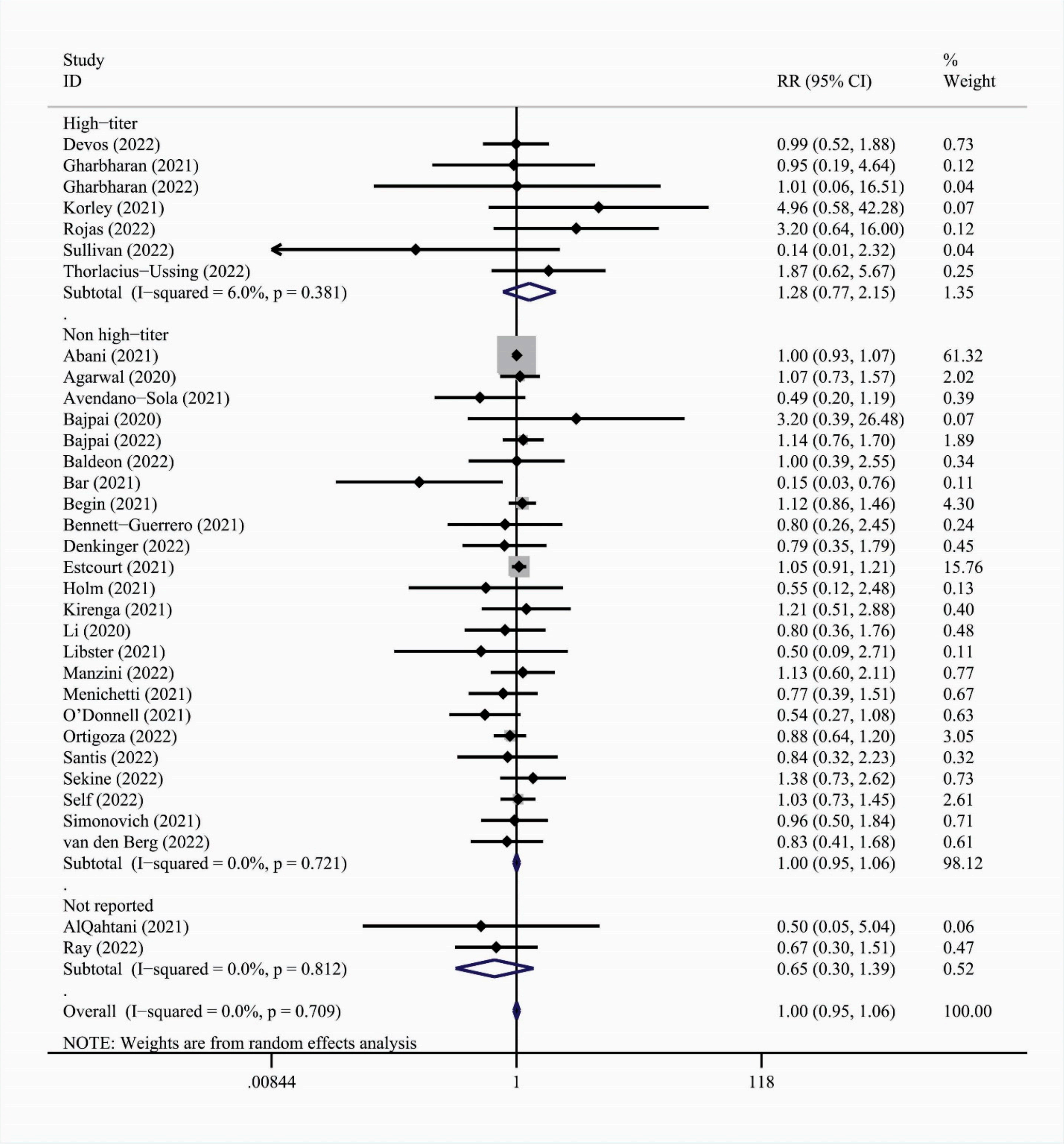

Figure S5. Forest plot describing the association between convalescent plasma treatment and 28-day mortality. Apart from the overall analysis, the sub-analysis by titer is presented.

Supplementary Figure S6. 28-day hospitalization, by adjustment

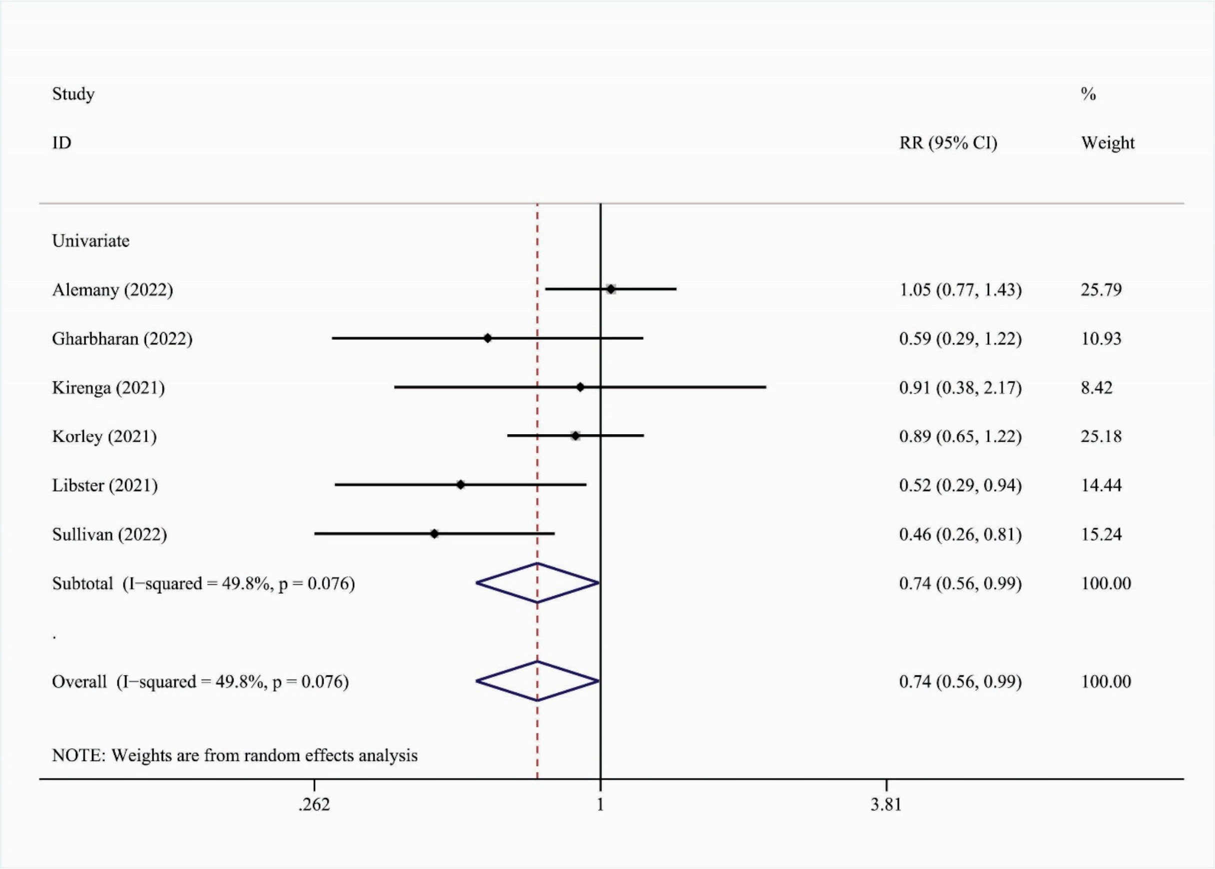

Figure S6. Forest plot describing the association between convalescent plasma treatment and 28-day hospitalization. Apart from the overall analysis, the sub-analysis by adjustment is presented.

Supplementary Figure S7. 28-day hospitalization, by multicenter status

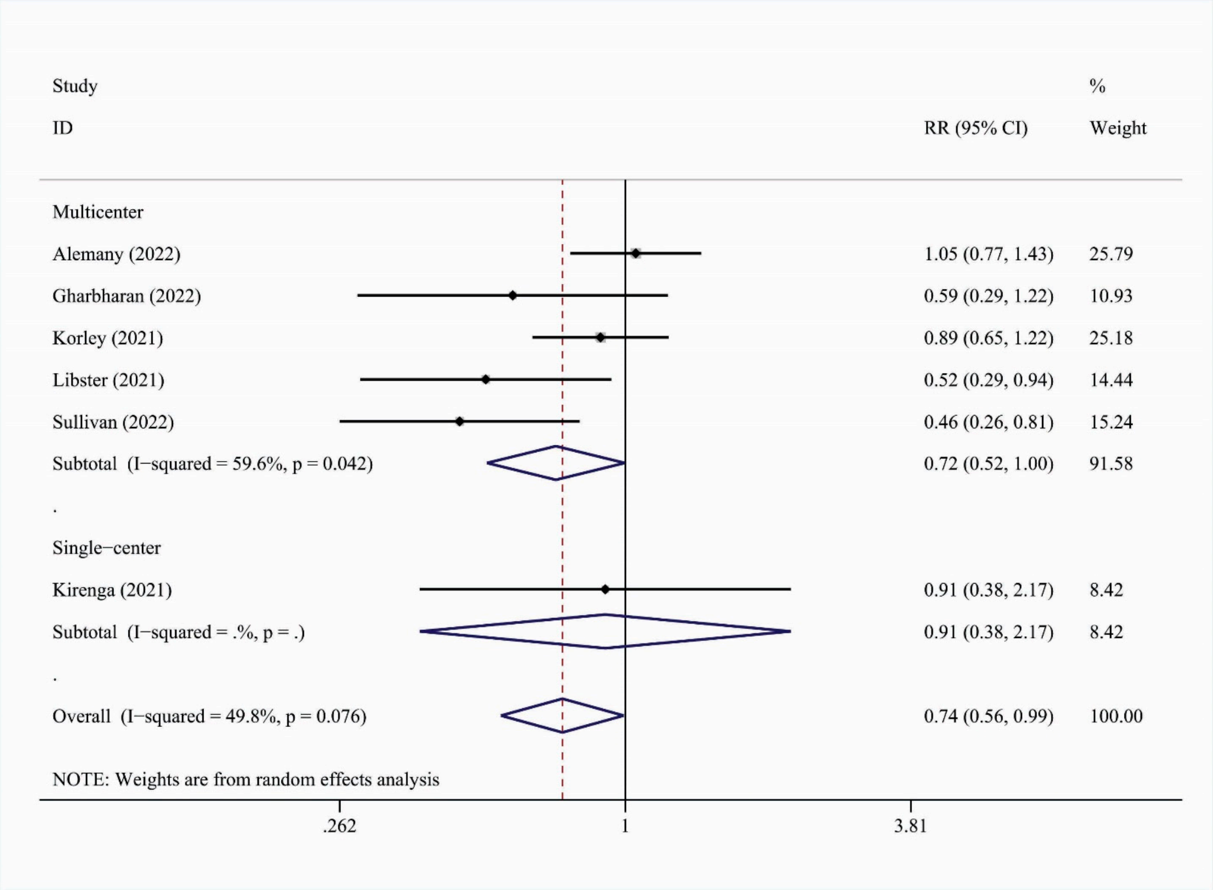

Figure S7. Forest plot describing the association between convalescent plasma treatment and 28-day hospitalization. Apart from the overall analysis, the sub-analysis by multicenter status is presented.

Supplementary Figure S8. 28-day hospitalization, by blinding status

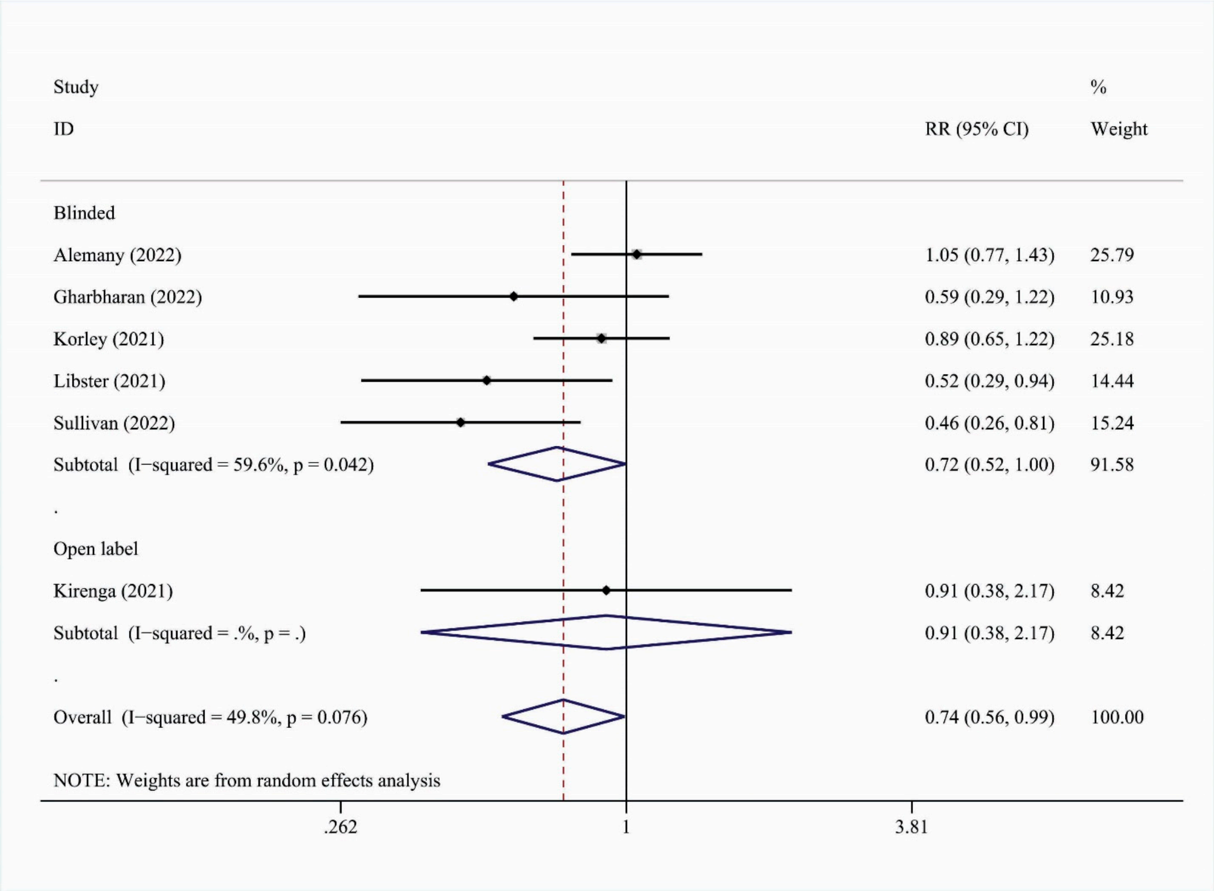

Figure S8. Forest plot describing the association between convalescent plasma treatment and 28-day hospitalization. Apart from the overall analysis, the sub-analysis by blinding status is presented.

Supplementary Figure S9. 28-day hospitalization, by geographic region

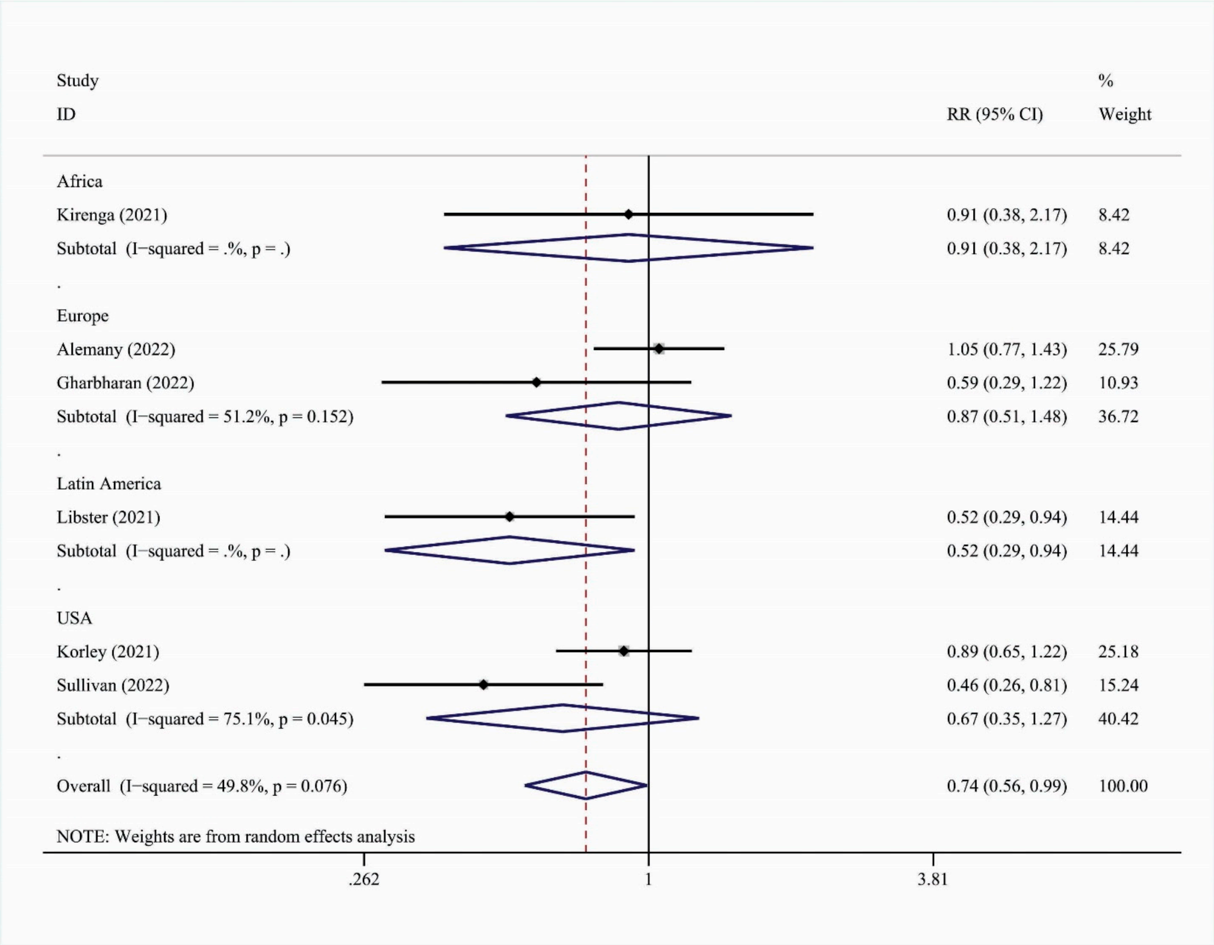

Figure S9. Forest plot describing the association between convalescent plasma treatment and 28-day hospitalization. Apart from the overall analysis, the sub-analysis by geographic region is presented.

Supplementary Figure S10. 28-day hospitalization, by titer

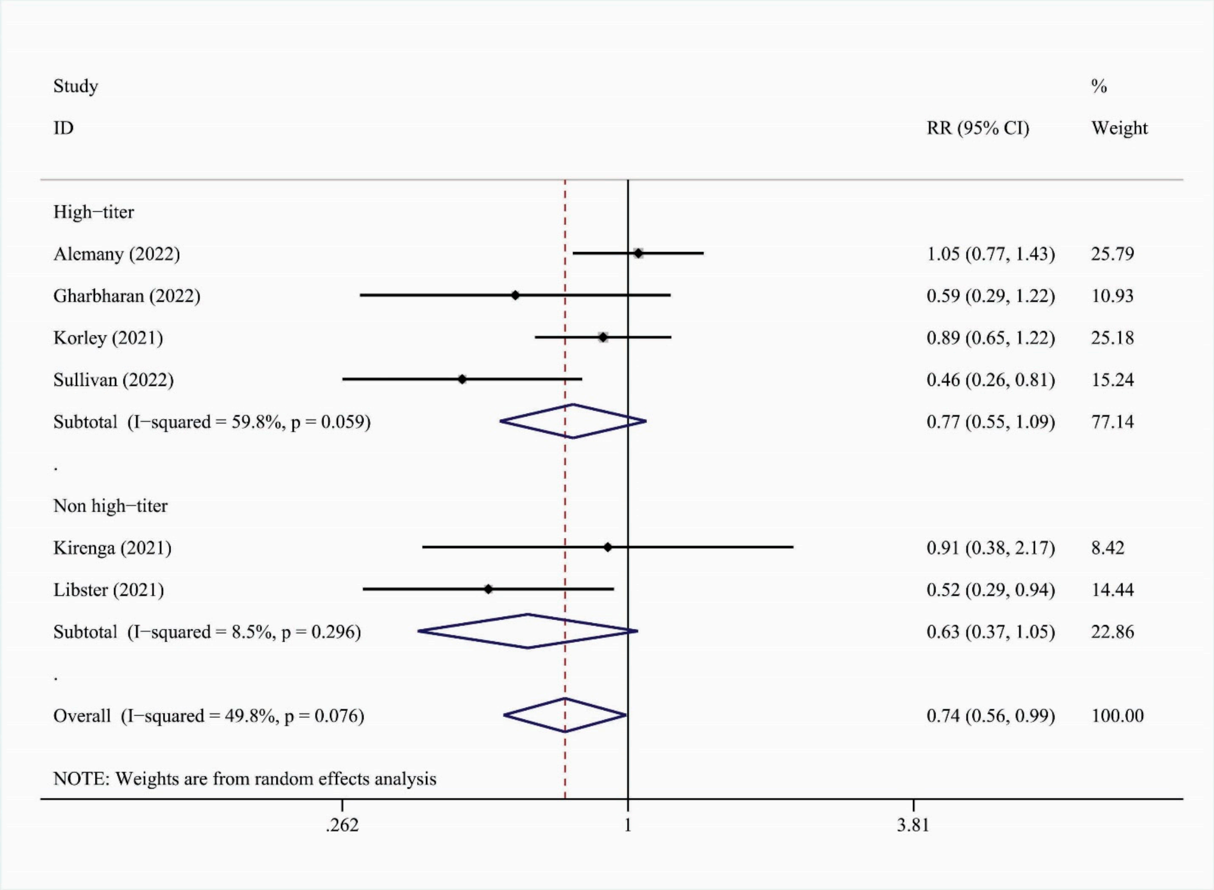

Figure S10. Forest plot describing the association between convalescent plasma treatment and 28-day hospitalization. Apart from the overall analysis, the sub-analysis by titer is presented.

Supplementary Figure S11. 28-day hospital discharge, by adjustment

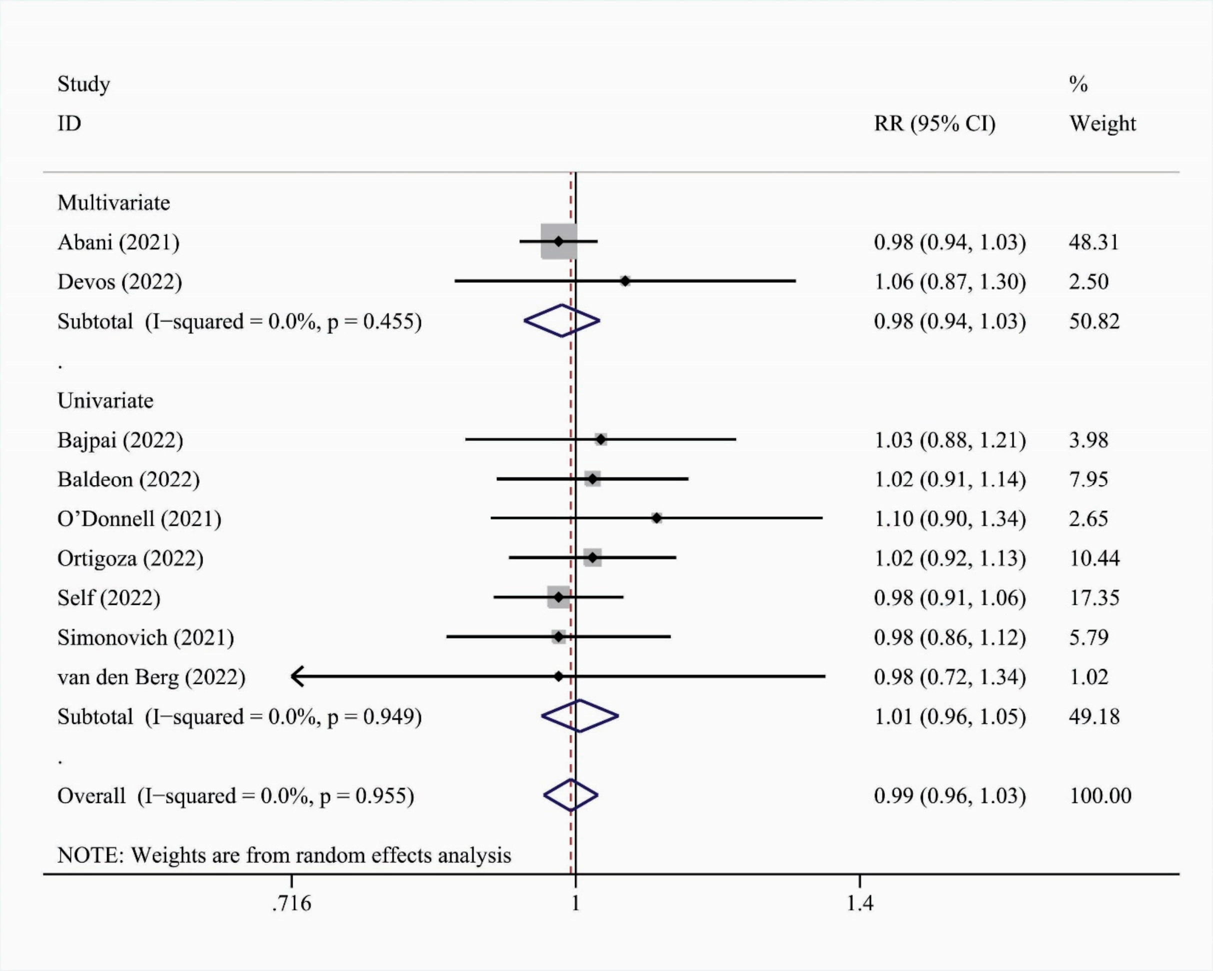

Figure S11. Forest plot describing the association between convalescent plasma treatment and 28-day hospital discharge. Apart from the overall analysis, the sub-analysis by adjustment is presented.

Supplementary Figure S12. 28-day hospital discharge, by multicenter status

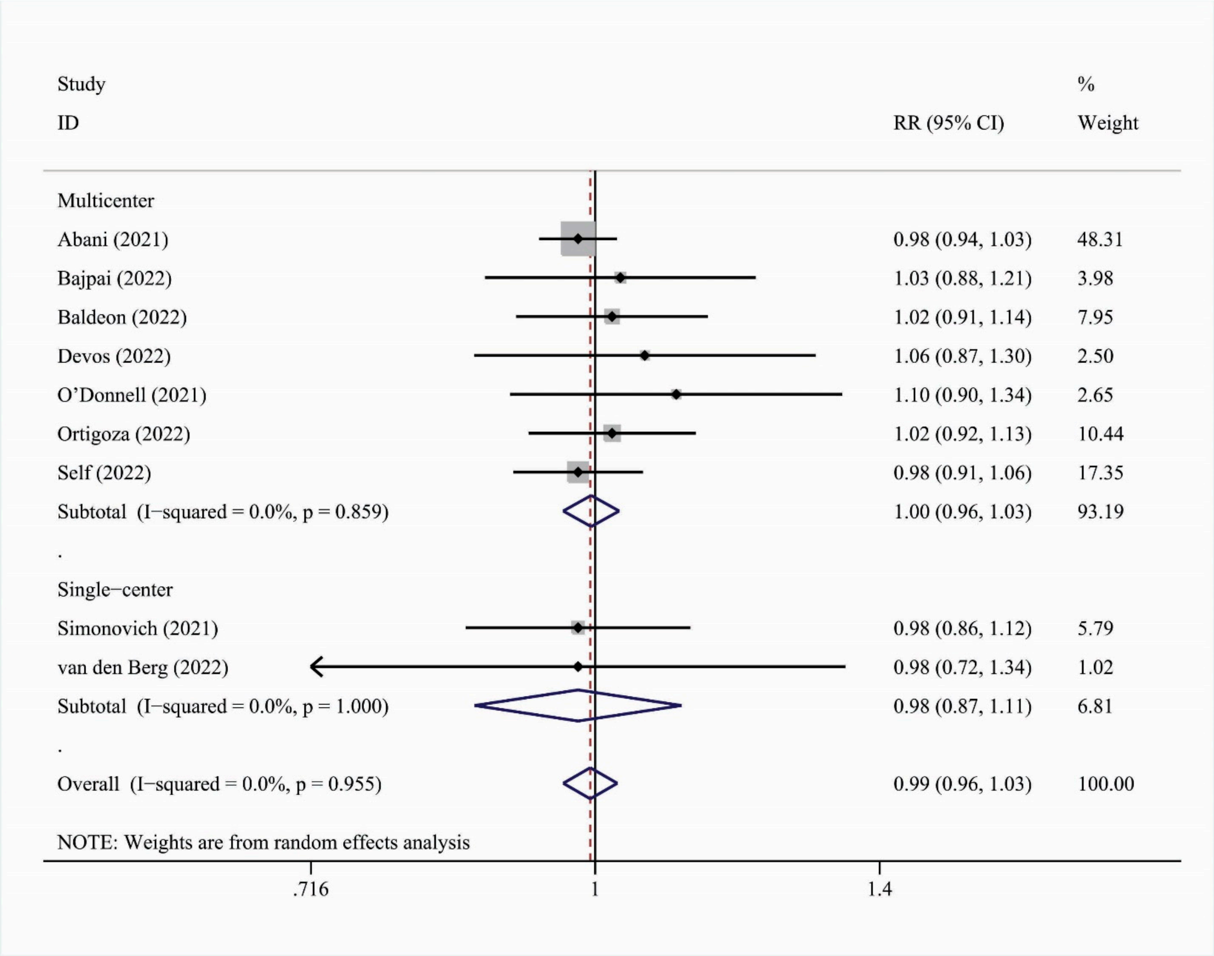

Figure S12. Forest plot describing the association between convalescent plasma treatment and 28-day hospital discharge. Apart from the overall analysis, the sub-analysis by multicenter is presented.

Supplementary Figure S13. 28-day hospital discharge, by blinding status

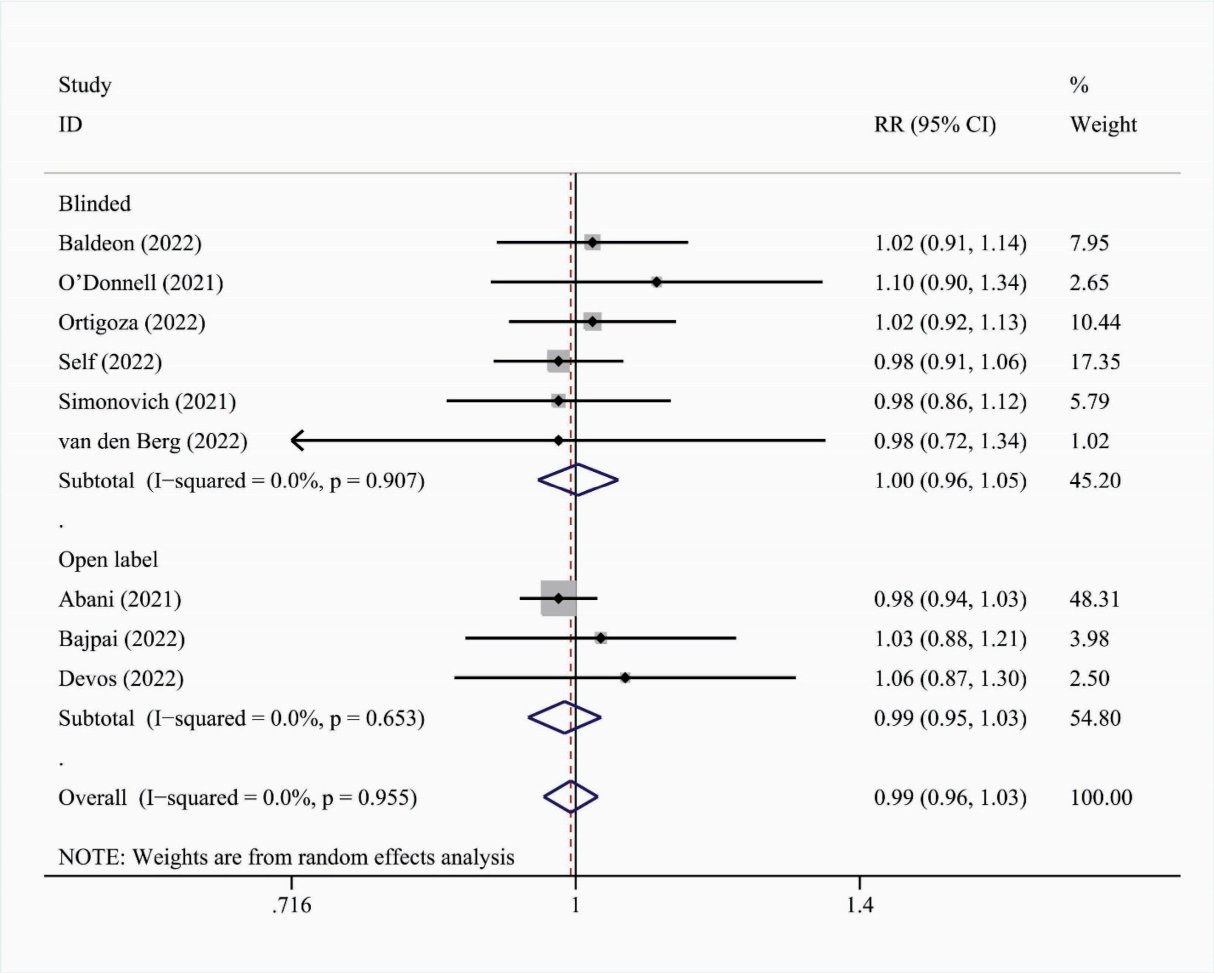

Figure S13. Forest plot describing the association between convalescent plasma treatment and 28-day hospital discharge. Apart from the overall analysis, the sub-analysis by blinding status is presented.

Supplementary Figure S14. 28-day hospital discharge, by geographic region

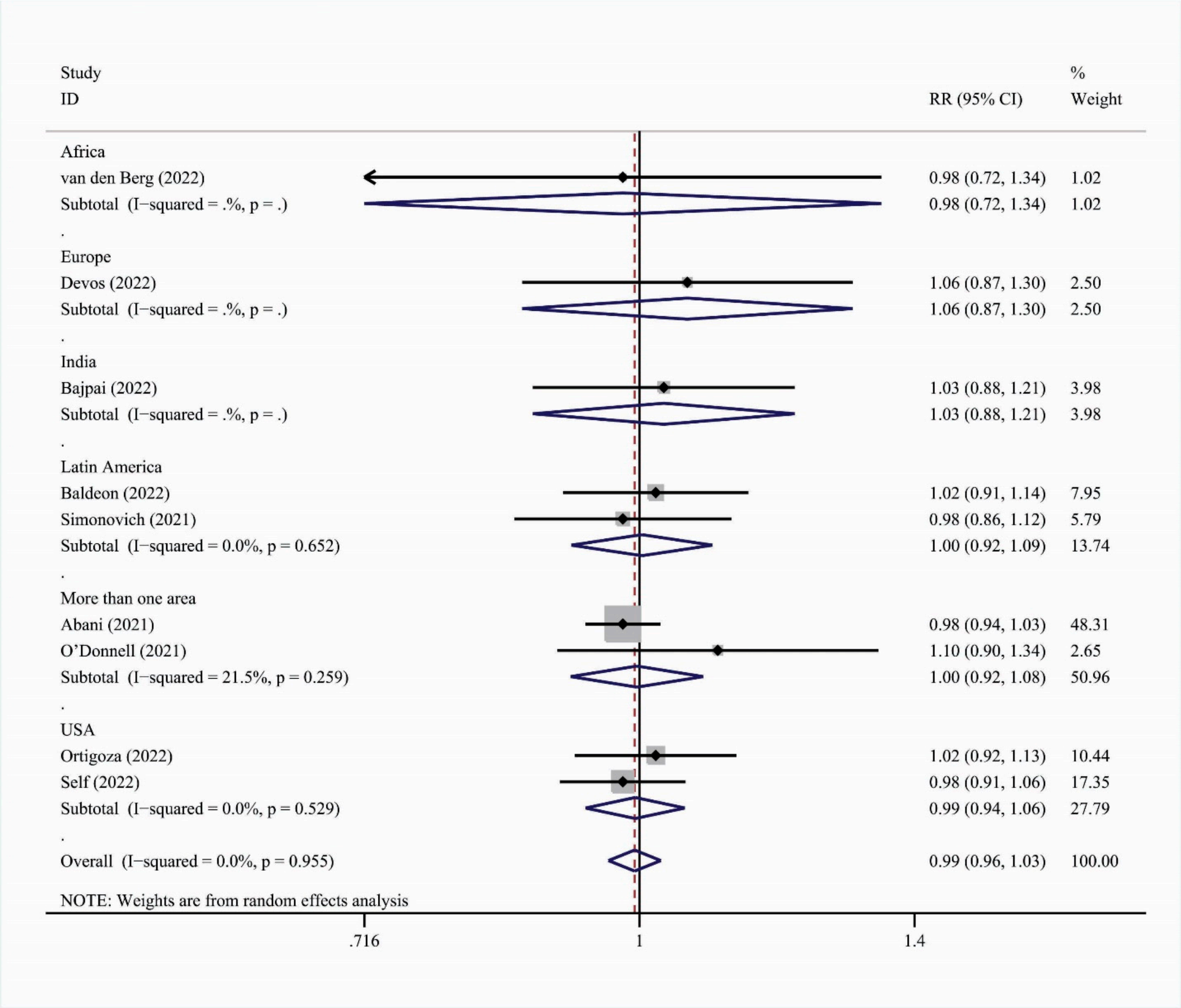

Figure S14. Forest plot describing the association between convalescent plasma treatment and 28-day hospital discharge. Apart from the overall analysis, the sub-analysis by geographic region is presented.

Supplementary Figure S15. 28-day hospital discharge, by titer

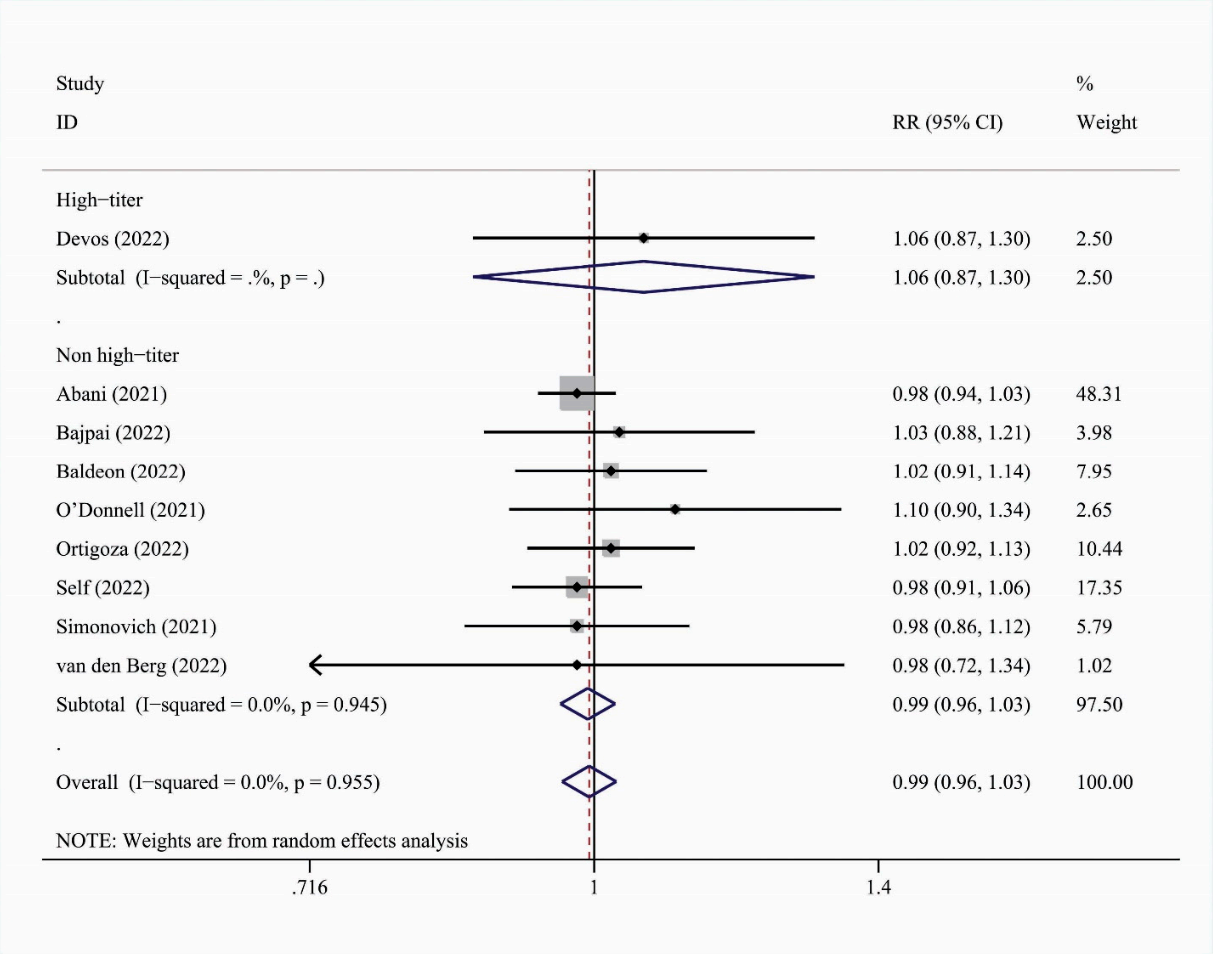

Figure S15. Forest plot describing the association between convalescent plasma treatment and 28-day hospital discharge. Apart from the overall analysis, the sub-analysis by titer is presented.

Supplementary Figure S16. 28-day ICU-related outcomes, by adjustment

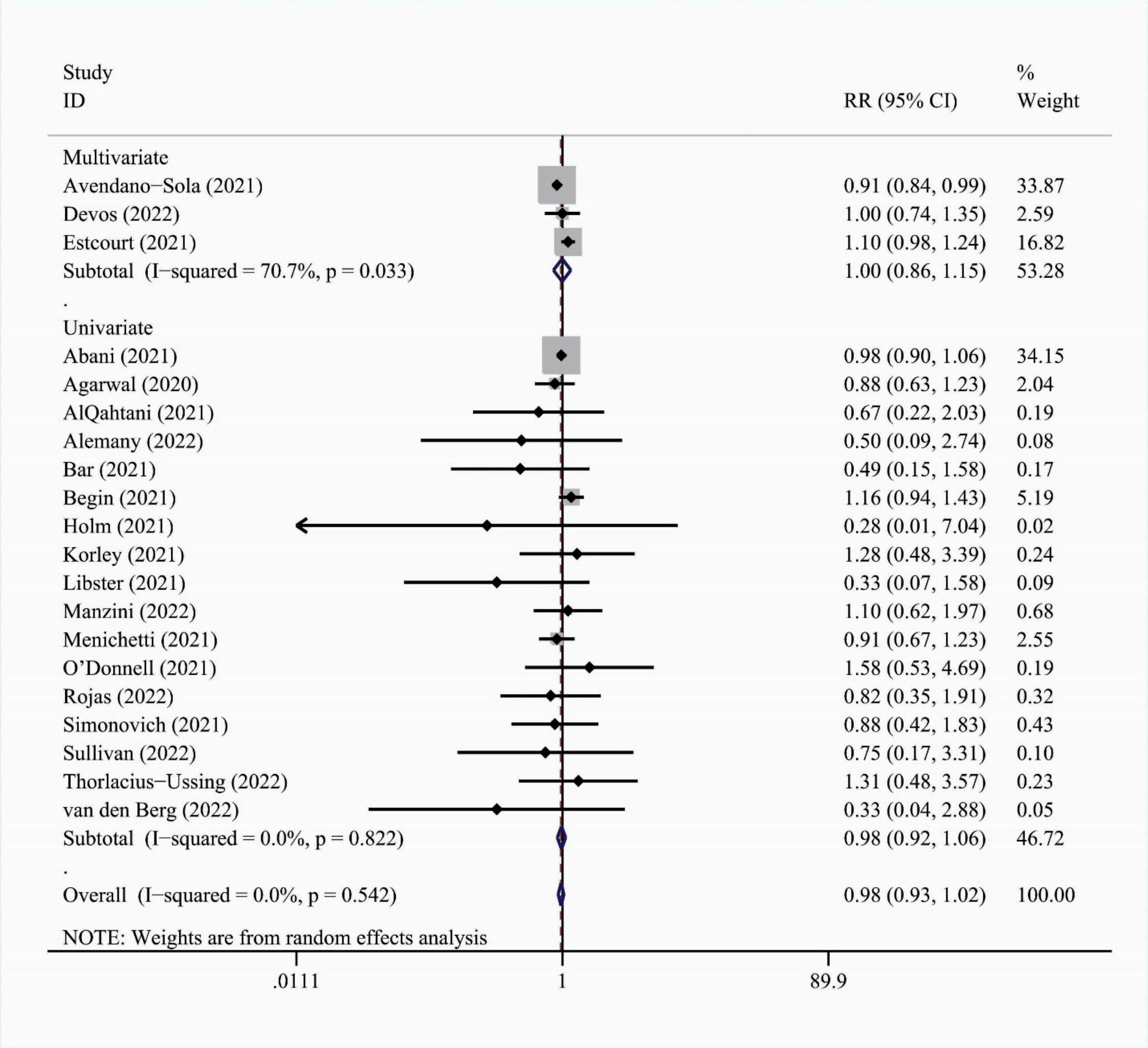

Figure S16. Forest plot describing the association between convalescent plasma treatment and 28-day ICU related outcomes. Apart from the overall analysis, the sub-analysis by adjustment is presented.

Supplementary Figure S17. 28-day ICU-related outcomes, by multicenter status

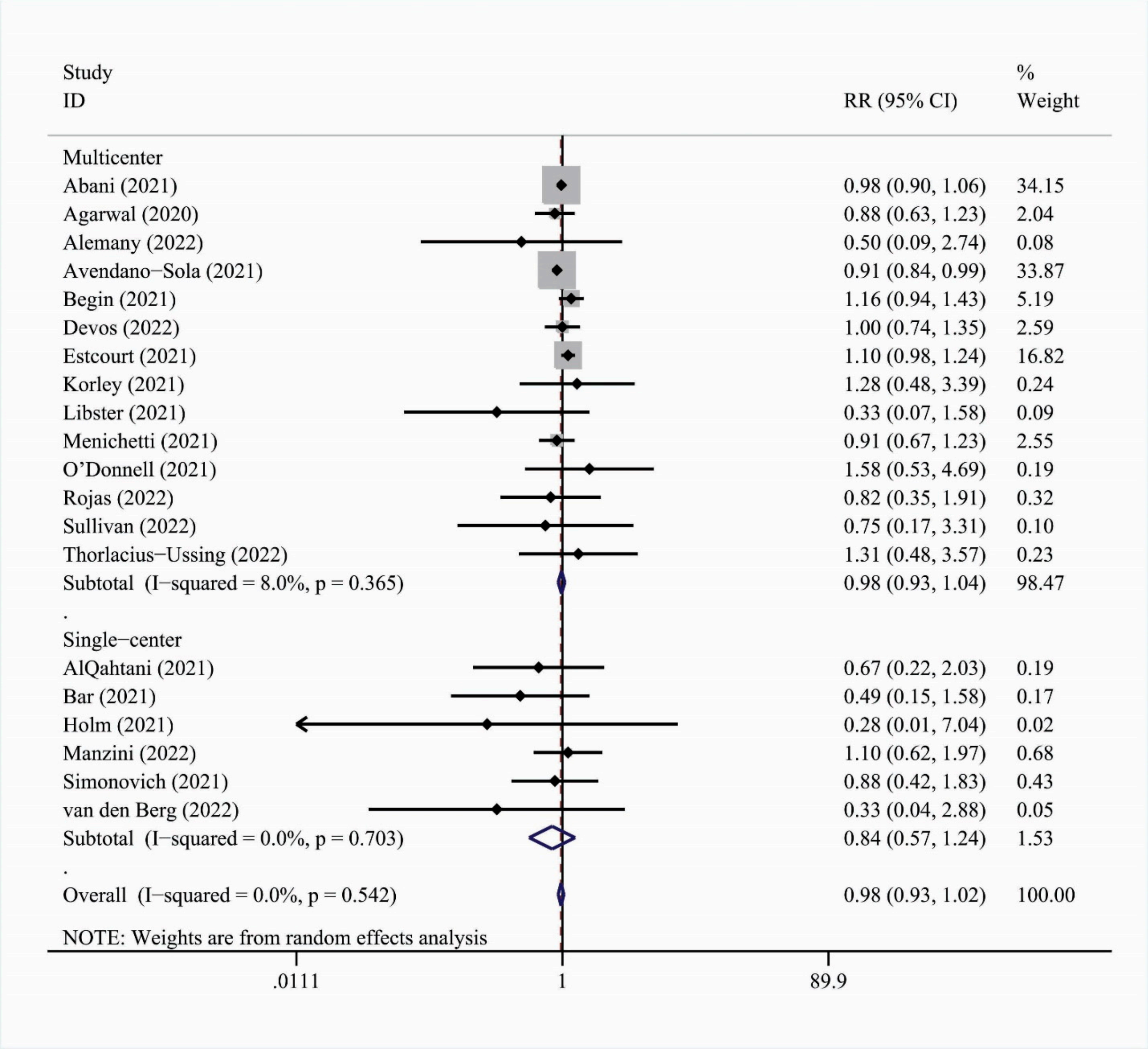

Figure S17. Forest plot describing the association between convalescent plasma treatment and 28-day ICU related outcomes. Apart from the overall analysis, the sub-analysis by multicenter status is presented.

Supplementary Figure S18. 28-day ICU-related outcomes, by adjustment

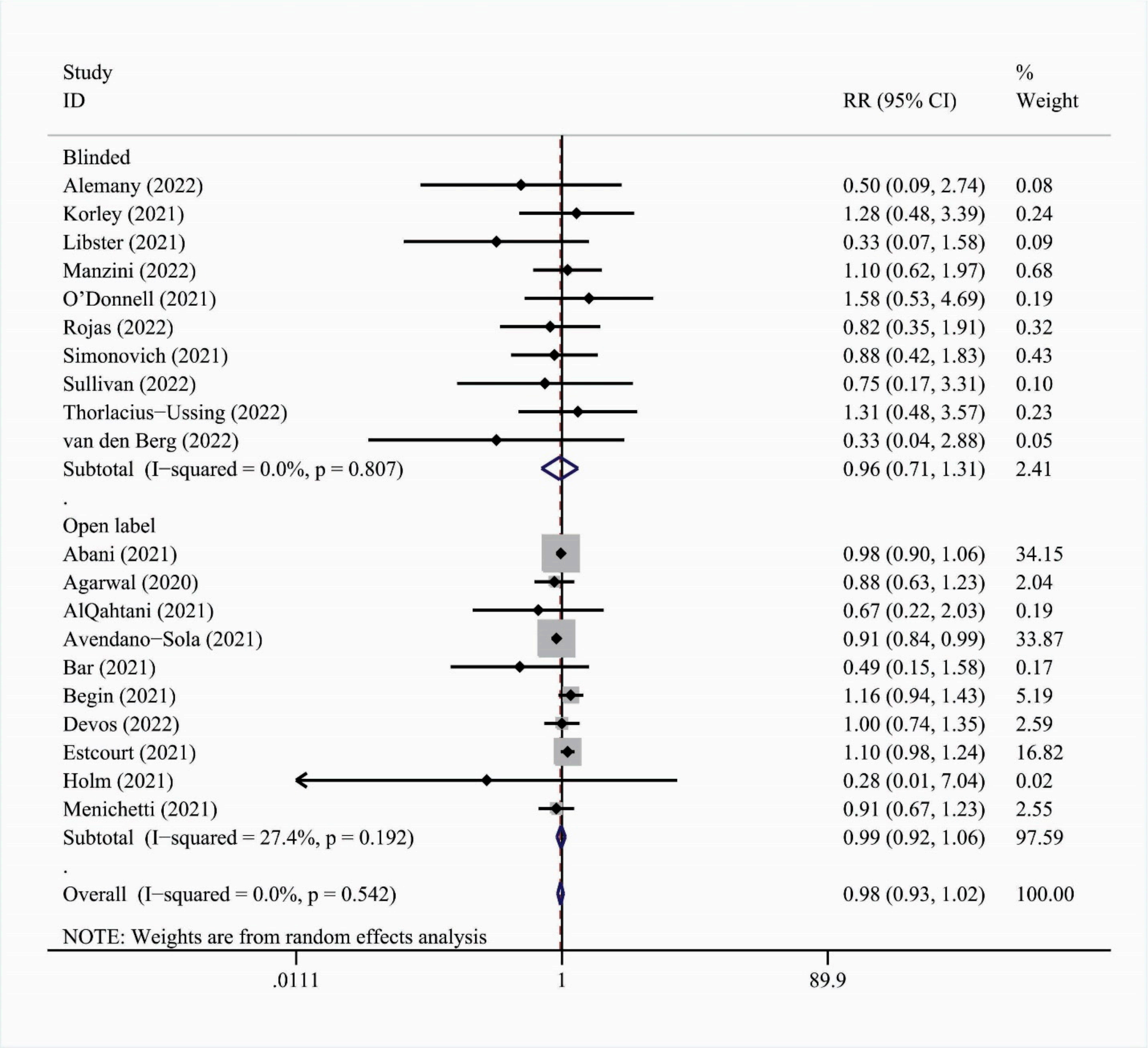

Figure S18. Forest plot describing the association between convalescent plasma treatment and 28-day ICU related outcomes. Apart from the overall analysis, the sub-analysis by blinding status is presented.

Supplementary Figure S19. 28-day ICU-related outcomes, by geographic region

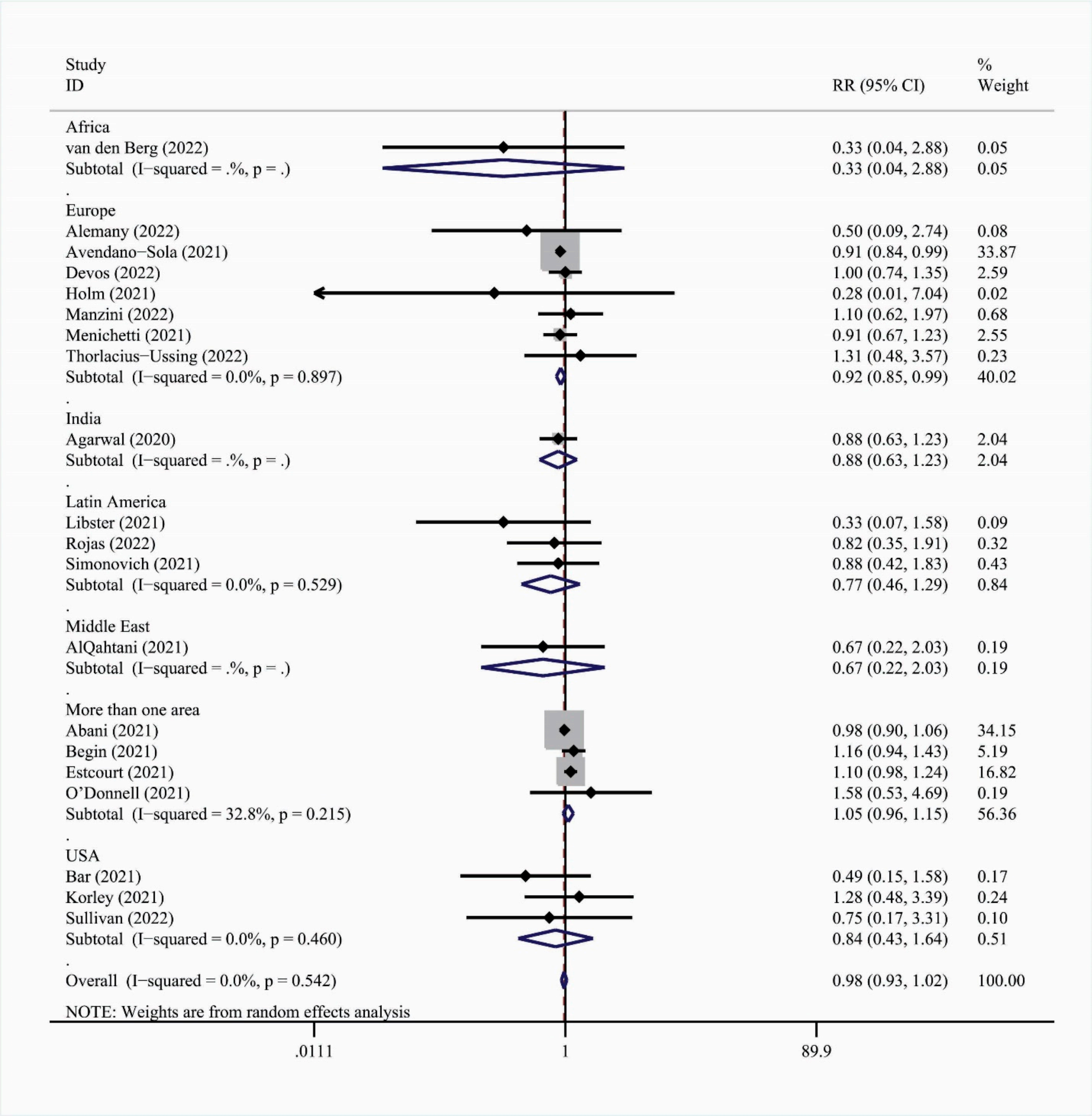

Figure S19. Forest plot describing the association between convalescent plasma treatment and 28-day ICU related outcomes. Apart from the overall analysis, the sub-analysis by geographic region is presented.

Supplementary Figure S20. 28-day ICU-related outcomes, by ICU status

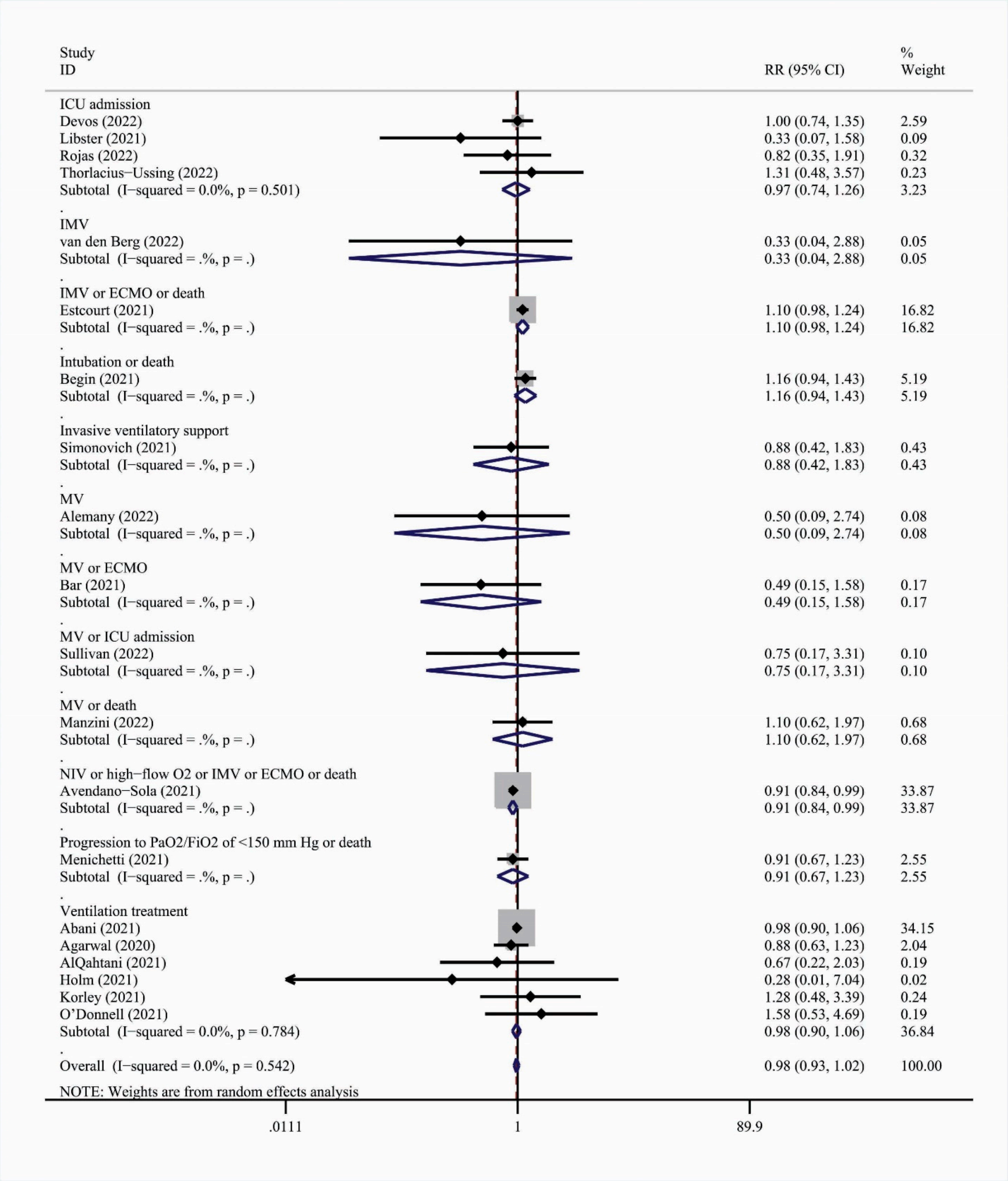

Figure S20. Forest plot describing the association between convalescent plasma treatment and 28-day ICU related outcomes. Apart from the overall analysis, the sub-analysis by ICU status is presented.

Supplementary Figure S21. 28-day ICU-related outcomes, by titer

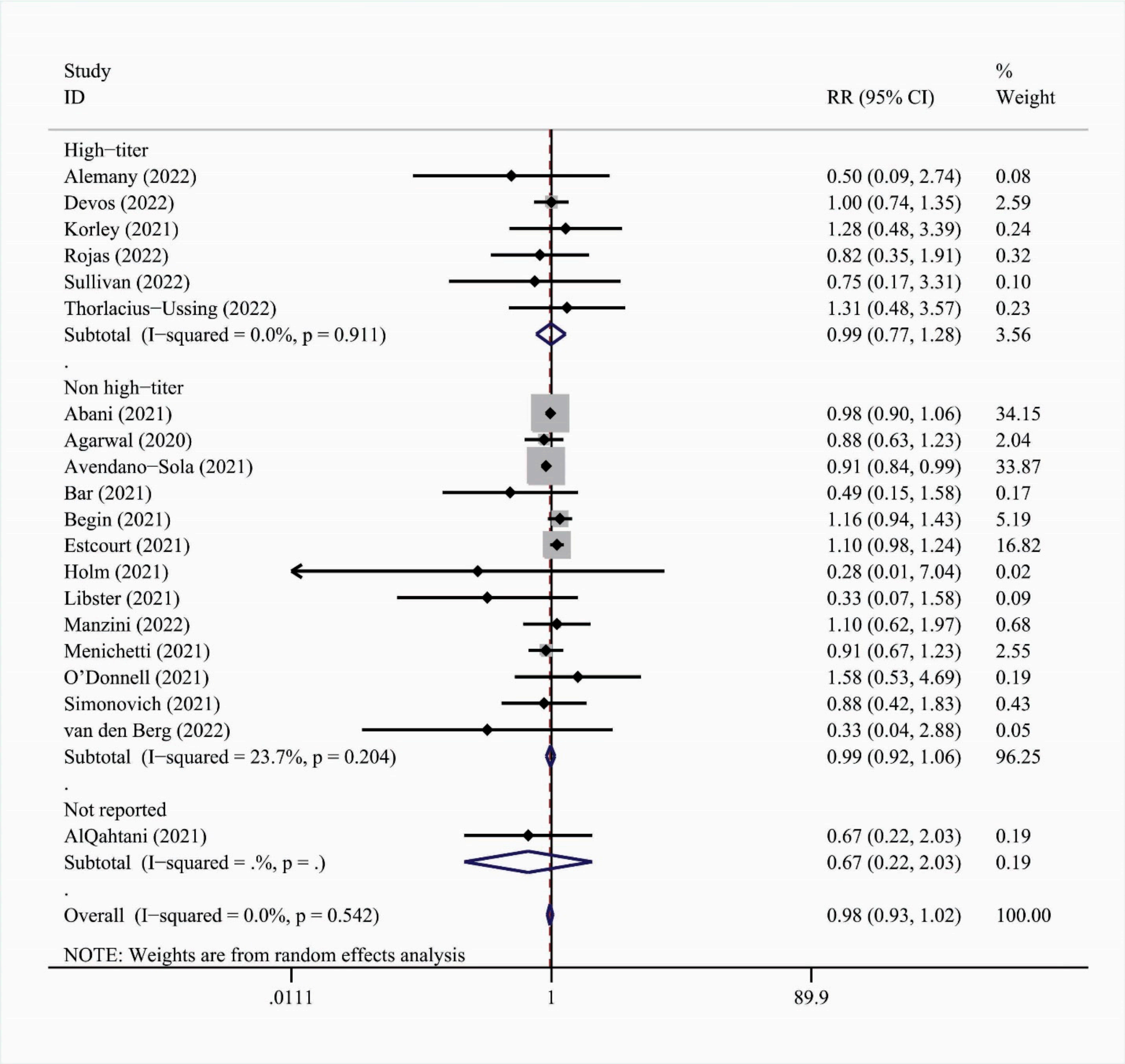

Figure S21. Forest plot describing the association between convalescent plasma treatment and 28-day ICU related outcomes. Apart from the overall analysis, the sub-analysis by titer is presented.

Supplementary Figure S22. 28-day score-related outcomes, by adjustment

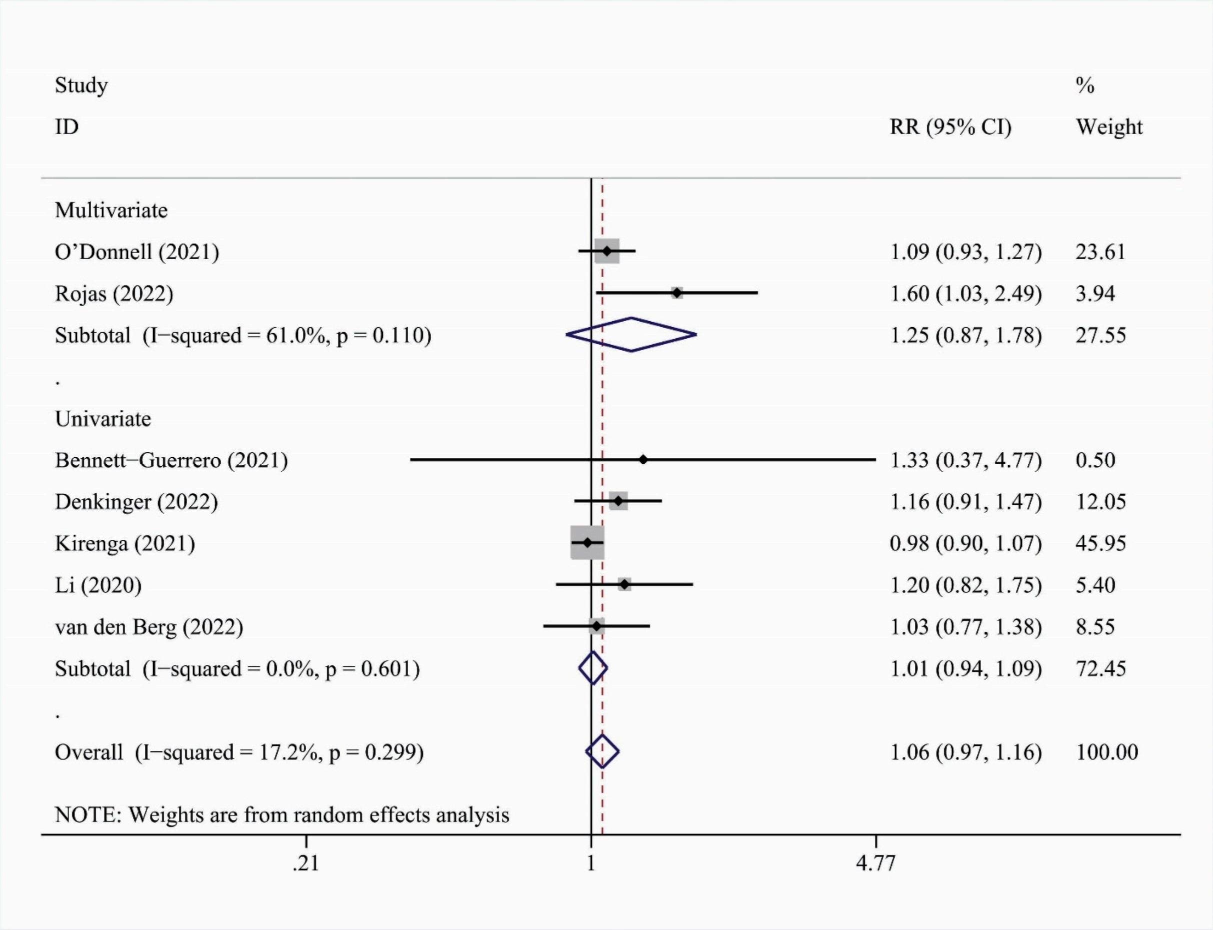

Figure S22. Forest plot describing the association between convalescent plasma treatment and 28-day score-related outcomes. Apart from the overall analysis, the sub-analysis by adjustment is presented.

Supplementary Figure S23. 28-day score-related outcomes, by multicenter status

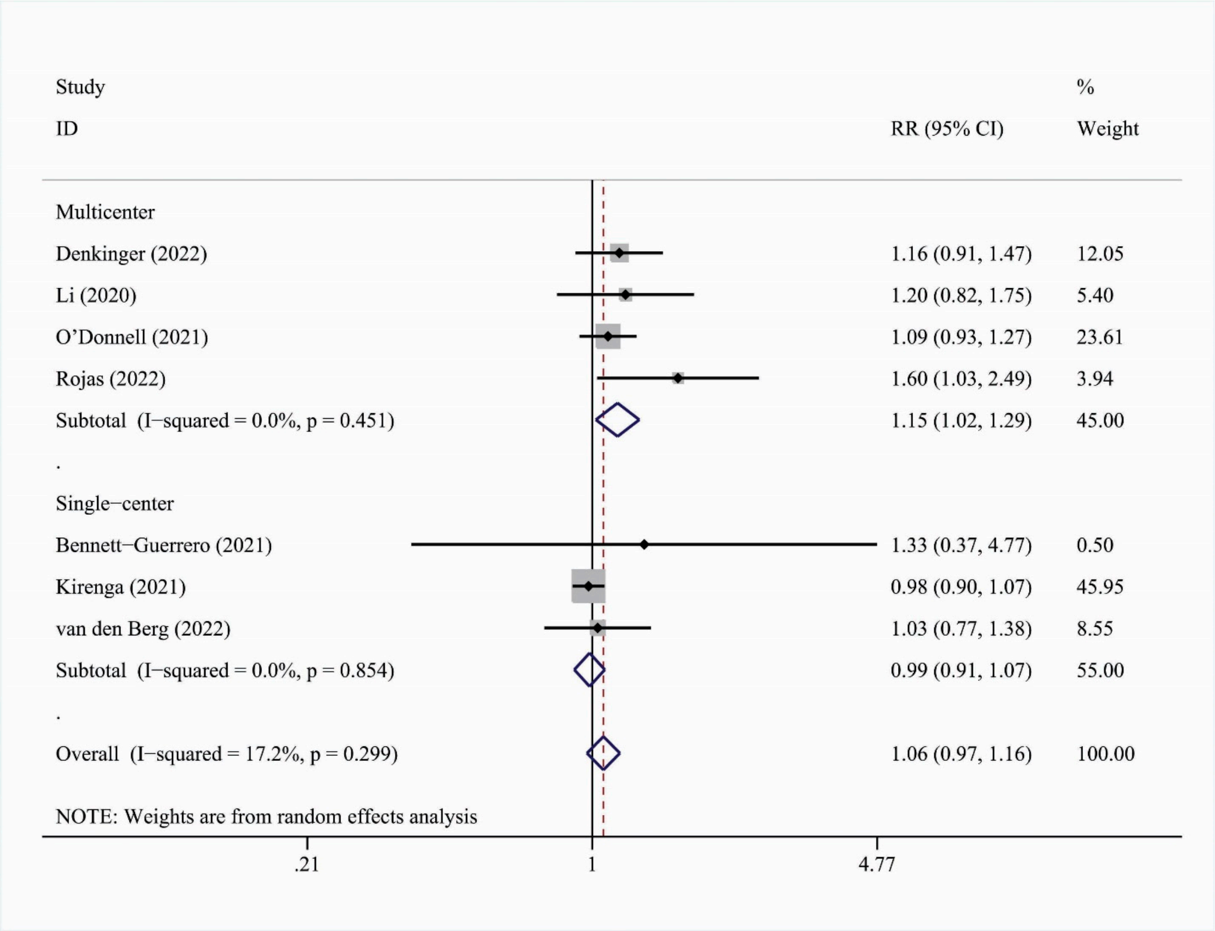

Figure S23. Forest plot describing the association between convalescent plasma treatment and 28-day score-related outcomes. Apart from the overall analysis, the sub-analysis by multicenter status is presented.

Supplementary Figure S24. 28-day score-related outcomes, by blinding status

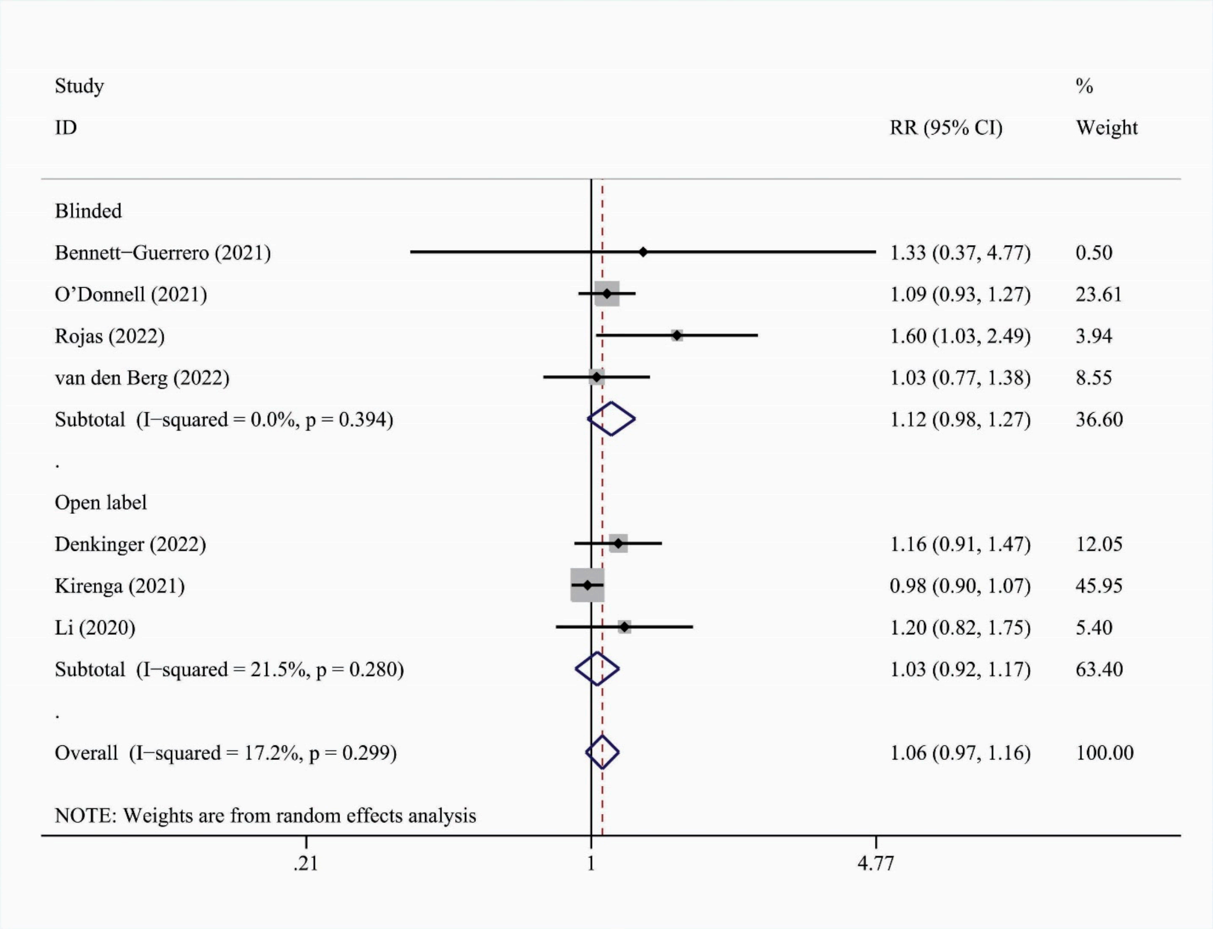

Figure S24. Forest plot describing the association between convalescent plasma treatment and 28-day score-related outcomes. Apart from the overall analysis, the sub-analysis by blinding status is presented.

Supplementary Figure S25. 28-day score-related outcomes, by geographic region

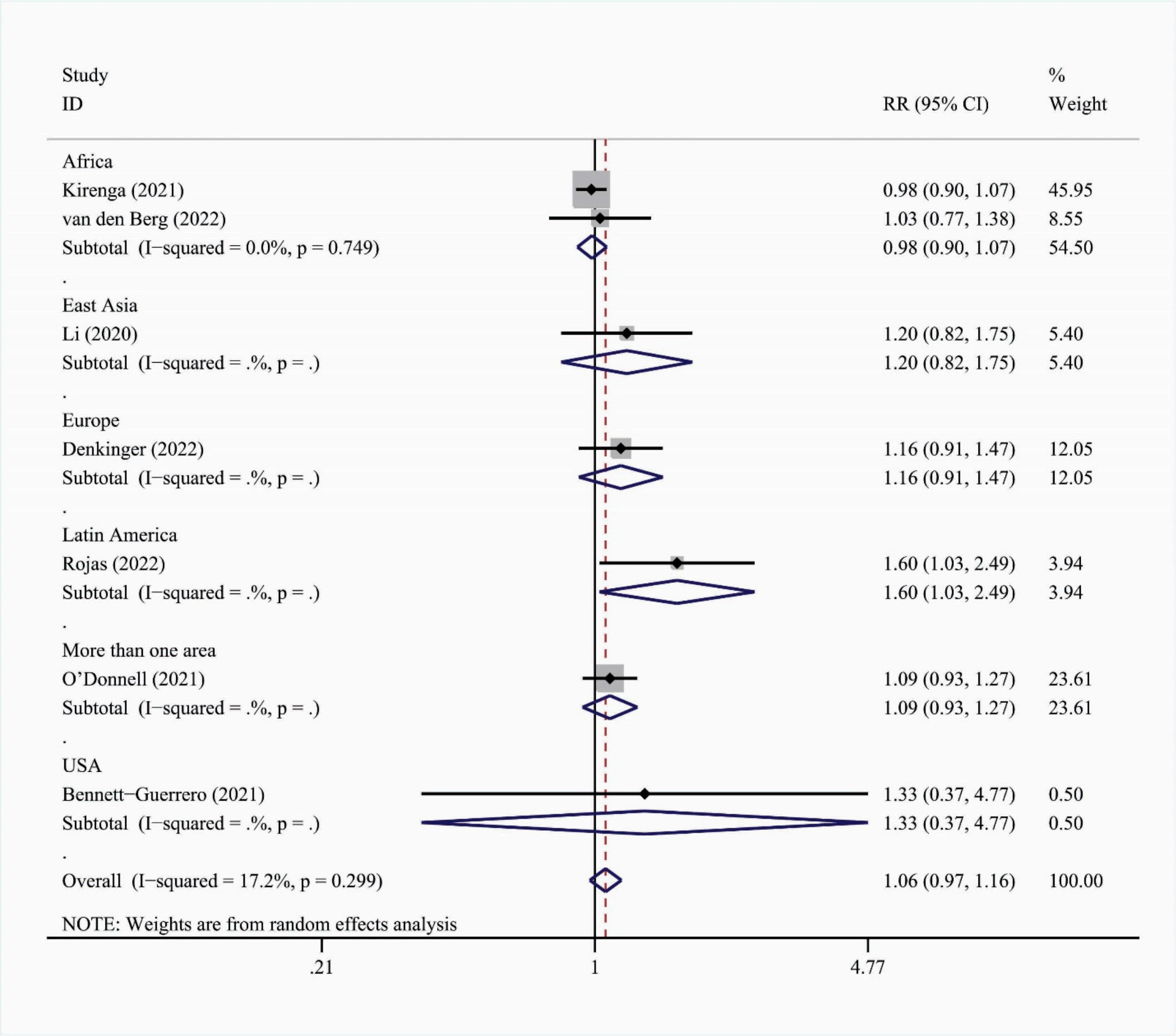

Figure S25. Forest plot describing the association between convalescent plasma treatment and 28-day score-related outcomes. Apart from the overall analysis, the sub-analysis by geographic region is presented.

Supplementary Figure S26. 28-day score-related outcomes, by titer

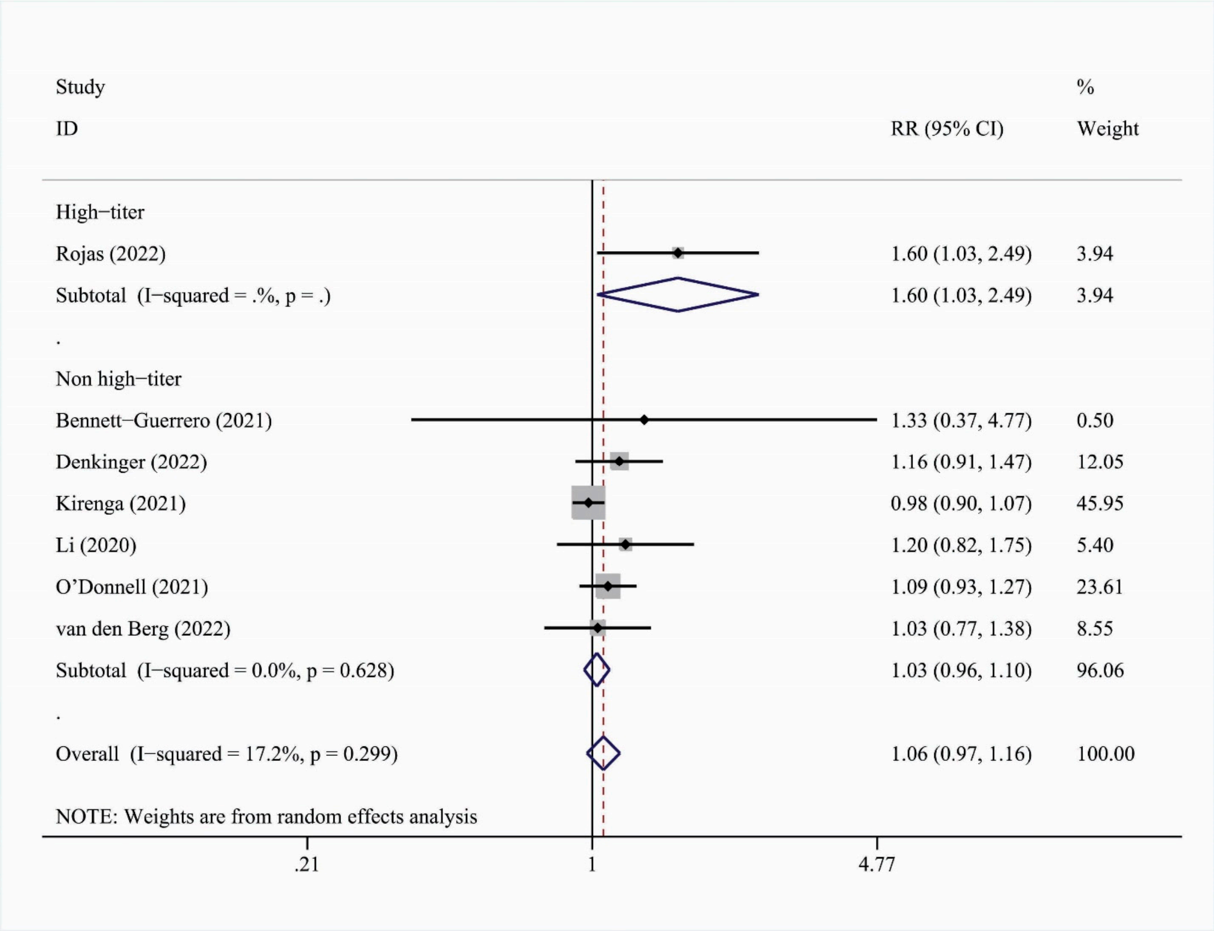

Figure S26. Forest plot describing the association between convalescent plasma treatment and 28-day score-related outcomes. Apart from the overall analysis, the sub-analysis by titer is presented.

Supplementary Figure S27. 14-day mortality, by adjustment

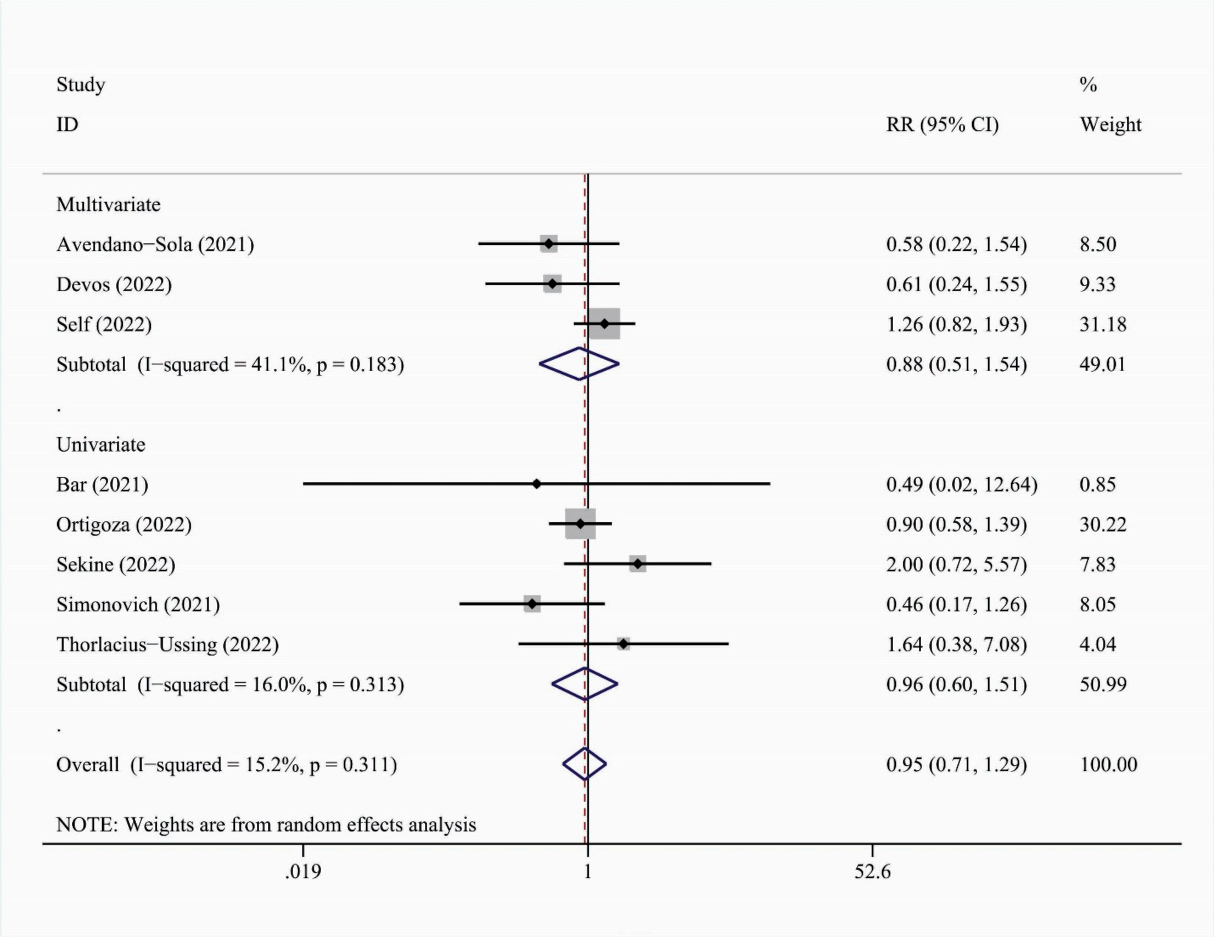

Figure S27. Forest plot describing the association between convalescent plasma treatment and 14-day mortality. Apart from the overall analysis, the sub-analysis by adjustment is presented.

Supplementary Figure S28. 14-day mortality, by multicenter status

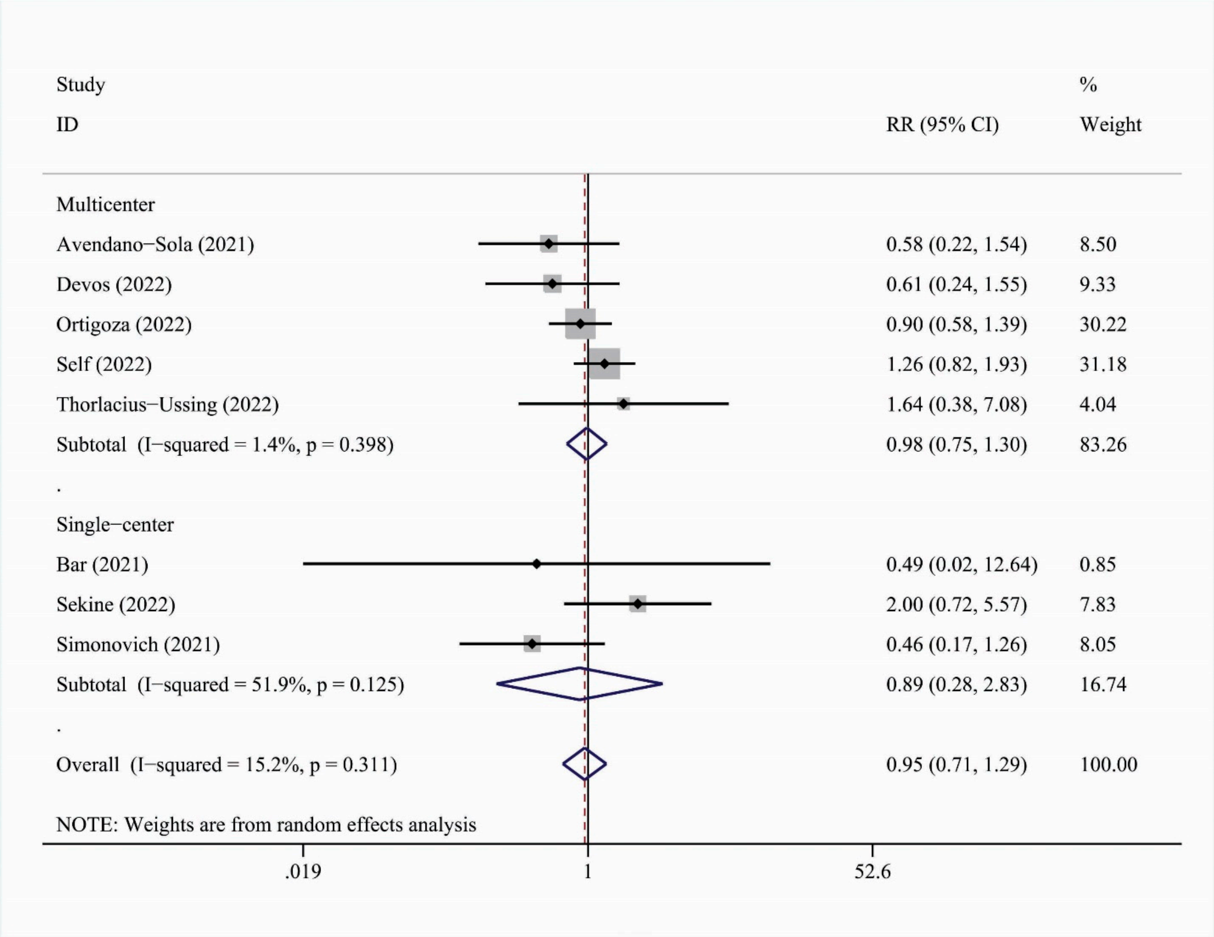

Figure S28. Forest plot describing the association between convalescent plasma treatment and 14-day mortality. Apart from the overall analysis, the sub-analysis by multicenter status is presented.

Supplementary Figure S29. 14-day mortality, by blinding status

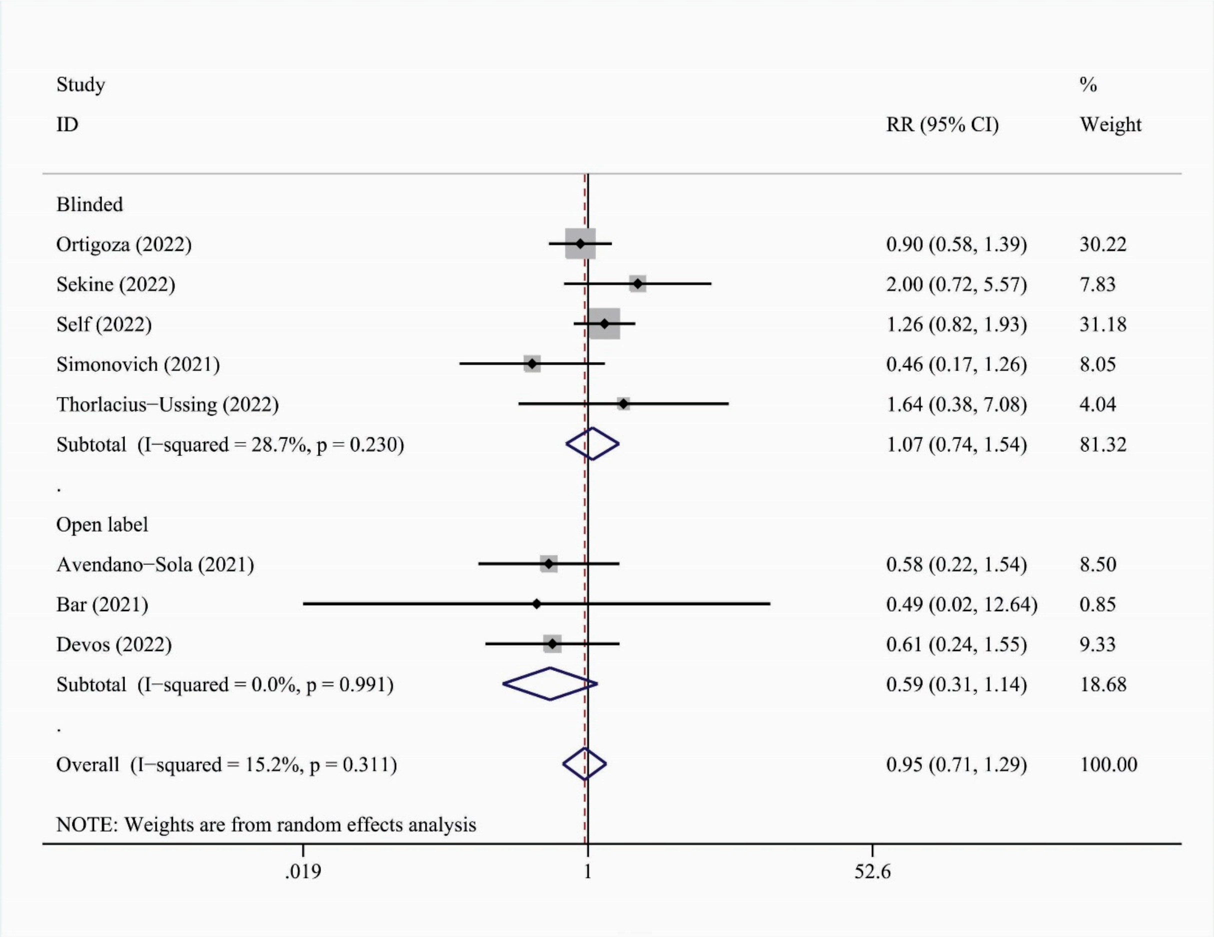

Figure S29. Forest plot describing the association between convalescent plasma treatment and 14-day mortality. Apart from the overall analysis, the sub-analysis by blinding status is presented.

Supplementary Figure S30. 14-day mortality, by geographic region

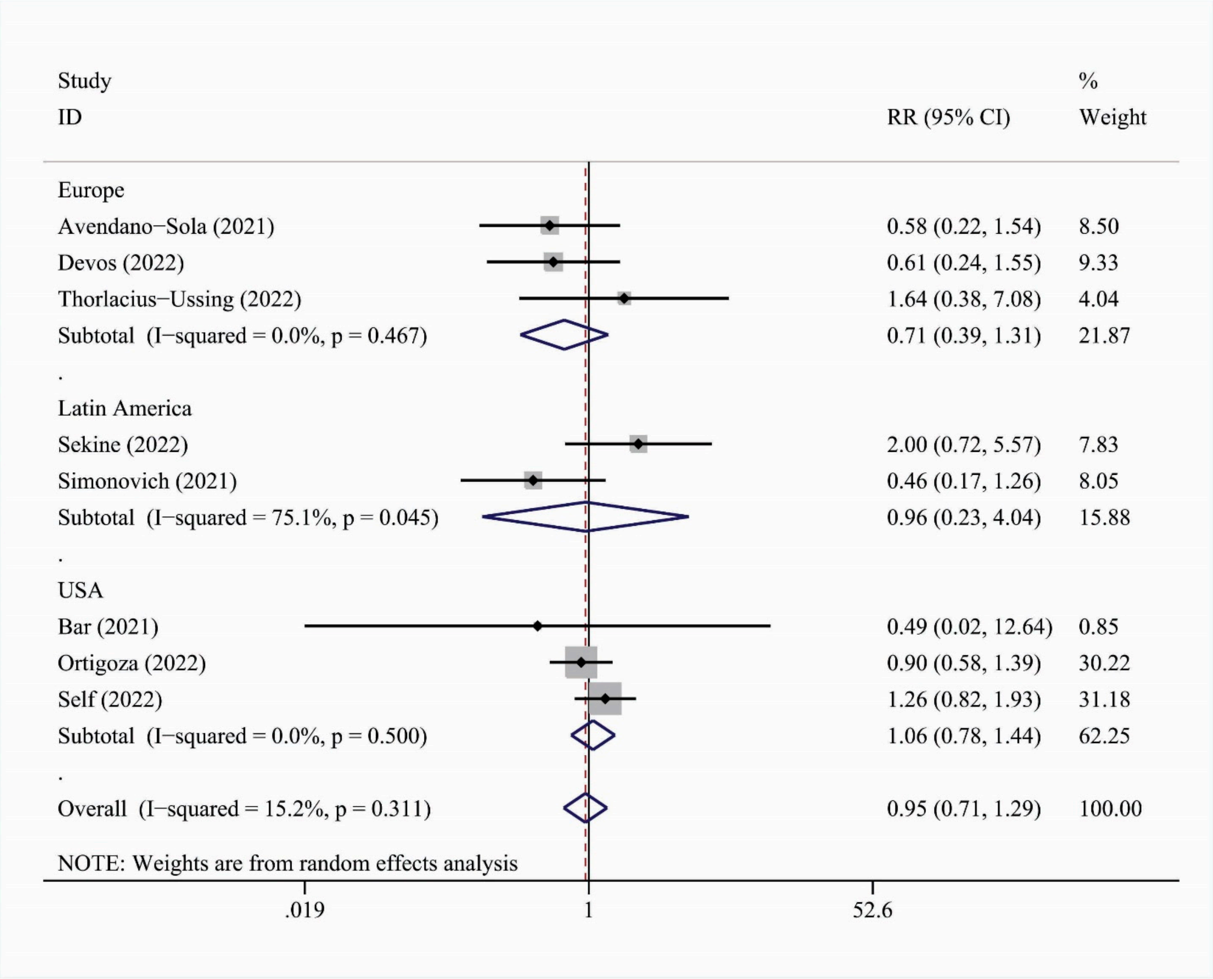

Figure S30. Forest plot describing the association between convalescent plasma treatment and 14-day mortality. Apart from the overall analysis, the sub-analysis by geographic region is presented.

Supplementary Figure S31. 14-day mortality, by titer

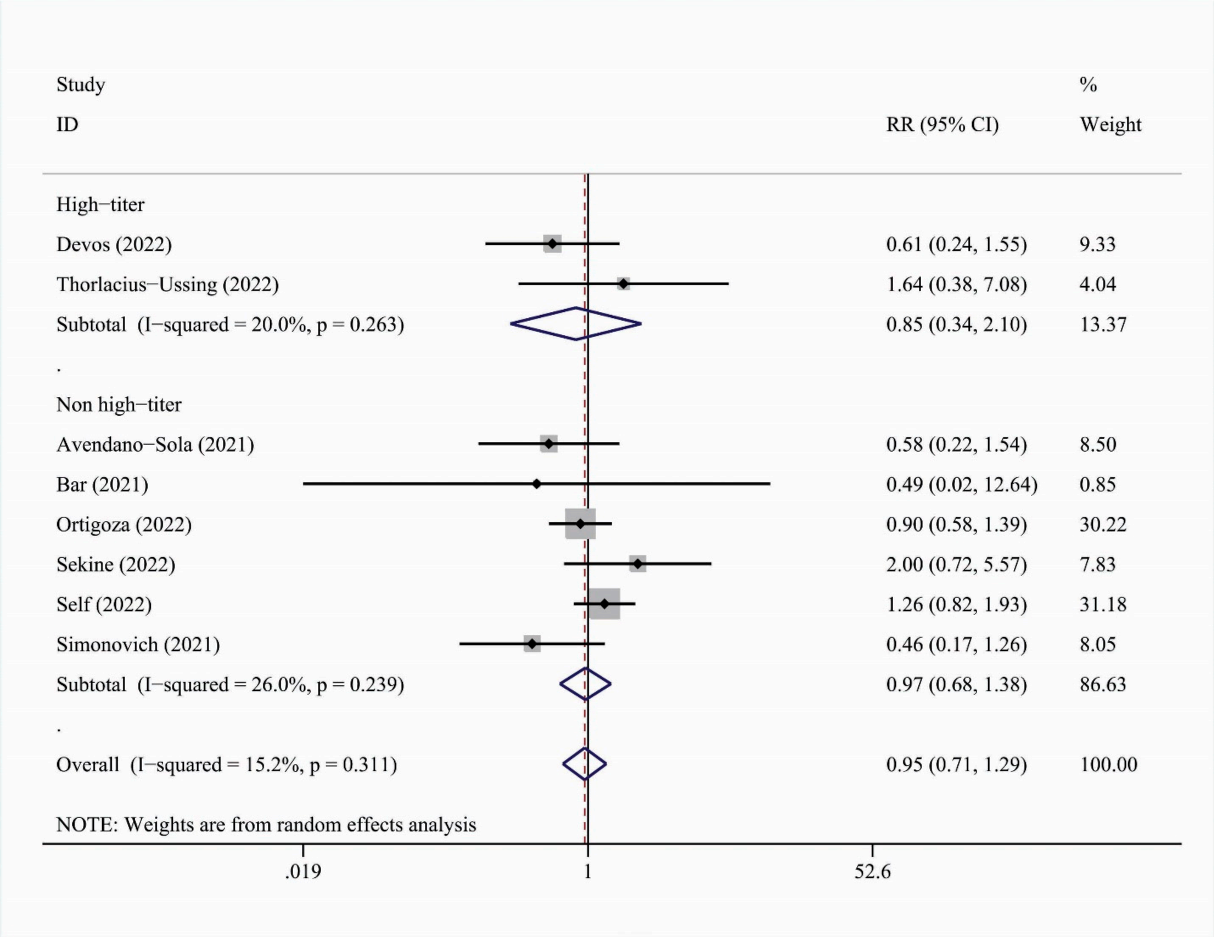

Figure S31. Forest plot describing the association between convalescent plasma treatment and 14-day mortality. Apart from the overall analysis, the sub-analysis titer is presented.

Supplementary Figure S32. 14-day hospital discharge, by adjustment

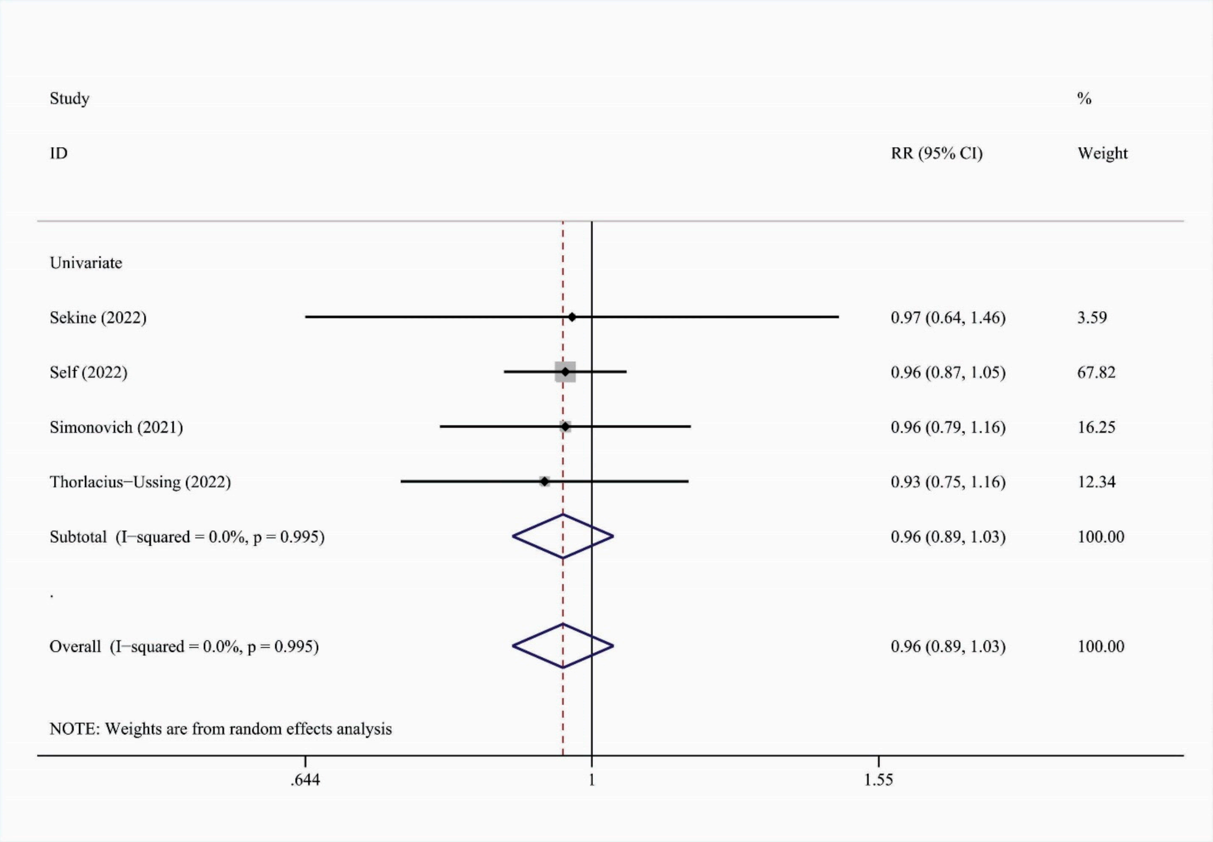

Figure S32. Forest plot describing the association between convalescent plasma treatment and 14-day hospital discharge. Apart from the overall analysis, the sub-analysis by adjustment is presented.

Supplementary Figure S33. 14-day hospital discharge, by multicenter status

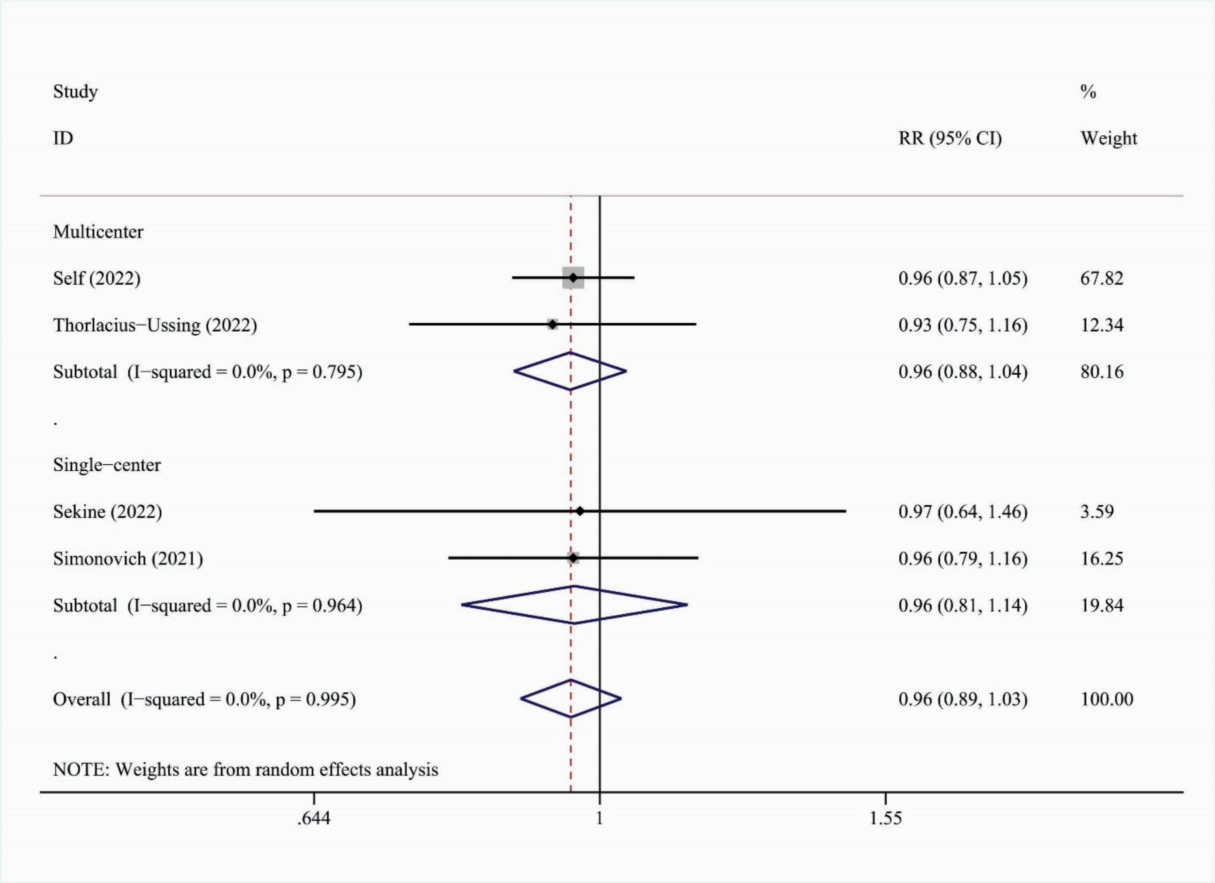

Figure S33. Forest plot describing the association between convalescent plasma treatment and 14-day hospital discharge. Apart from the overall analysis, the sub-analysis by multicenter status is presented.

Supplementary Figure S34. 14-day hospital discharge, by blinding status

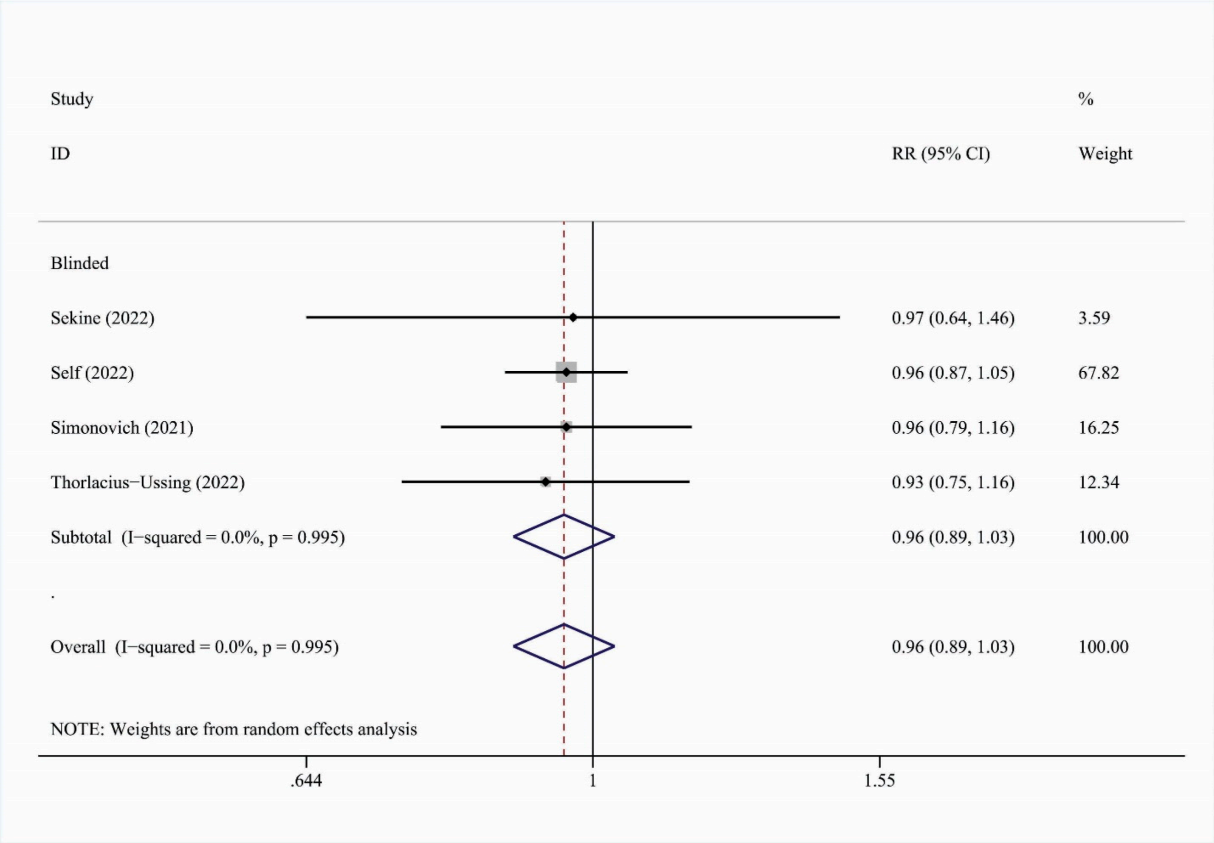

Figure S34. Forest plot describing the association between convalescent plasma treatment and 14-day hospital discharge. Apart from the overall analysis, the sub-analysis by blinding status is presented.

Supplementary Figure S35. 14-day hospital discharge, by multicenter status

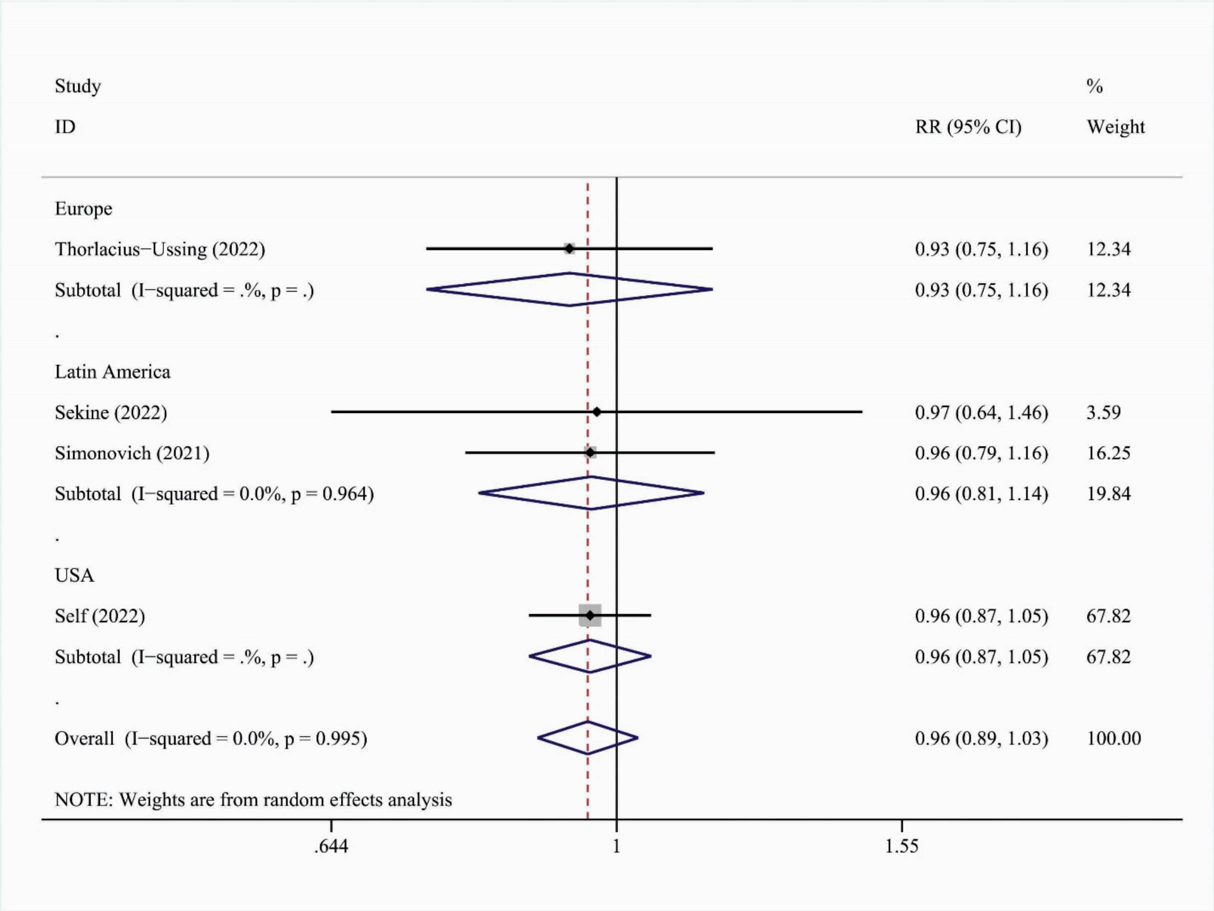

Figure S35. Forest plot describing the association between convalescent plasma treatment and 14-day hospital discharge. Apart from the overall analysis, the sub-analysis by geographic region is presented.

Supplementary Figure S36. 14-day hospital discharge, by titer

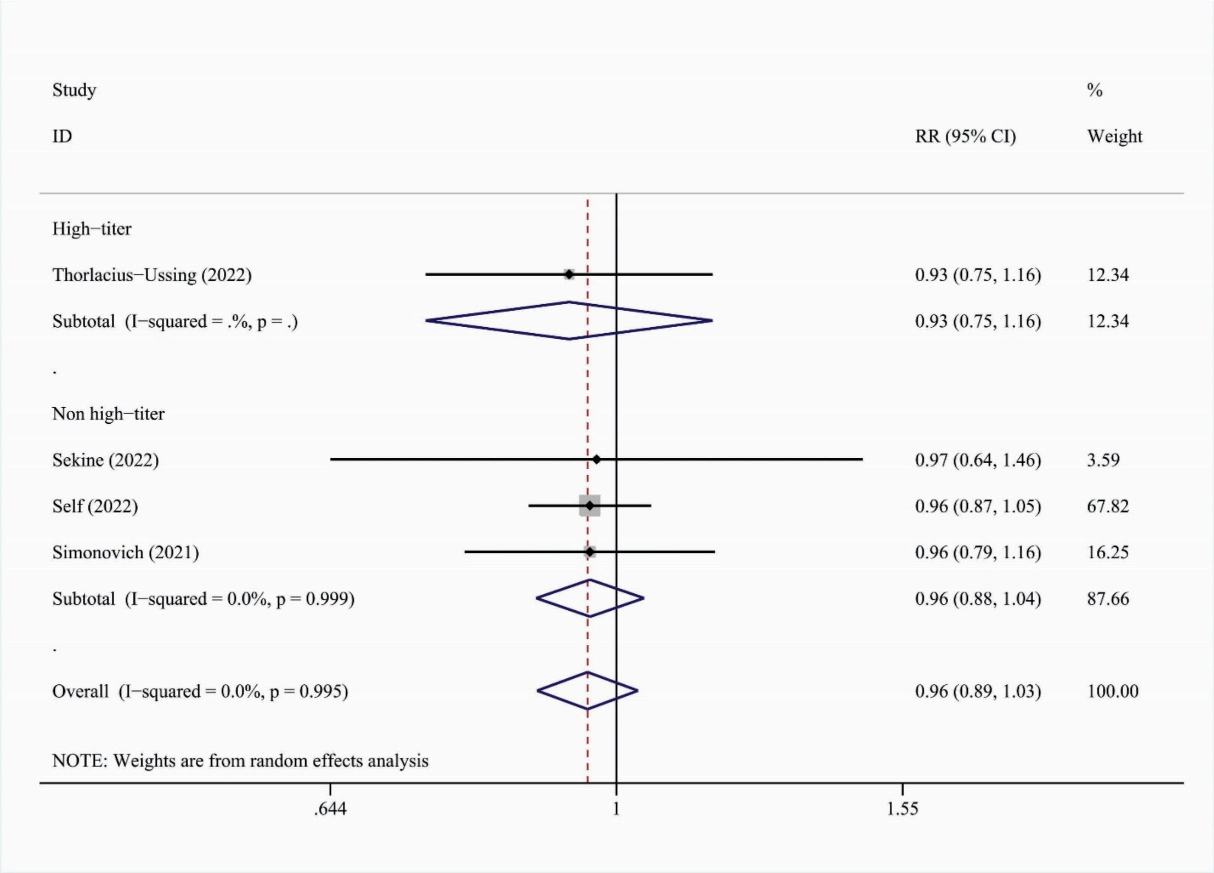

Figure S36. Forest plot describing the association between convalescent plasma treatment and 14-day hospital discharge. Apart from the overall analysis, the sub-analysis by titer is presented.

Supplementary Figure S37. Funnel plot, portraying publication bias for 28-day mortality

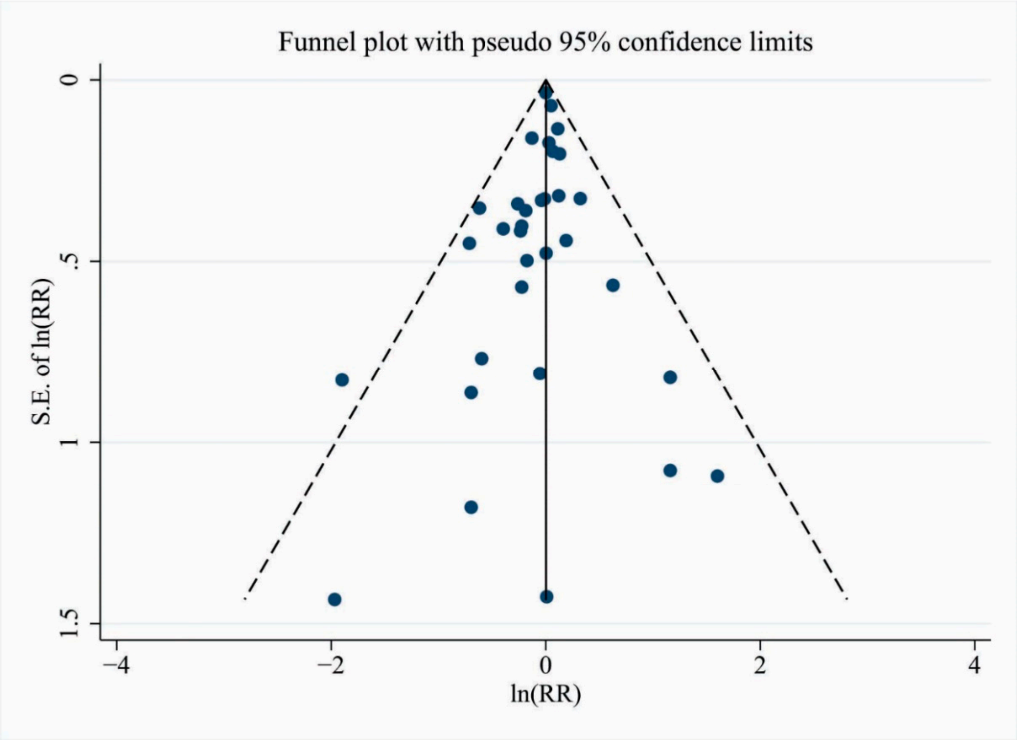

Supplementary Figure S38. Funnel plot, portraying publication bias for 28-day ICU-related outcomes

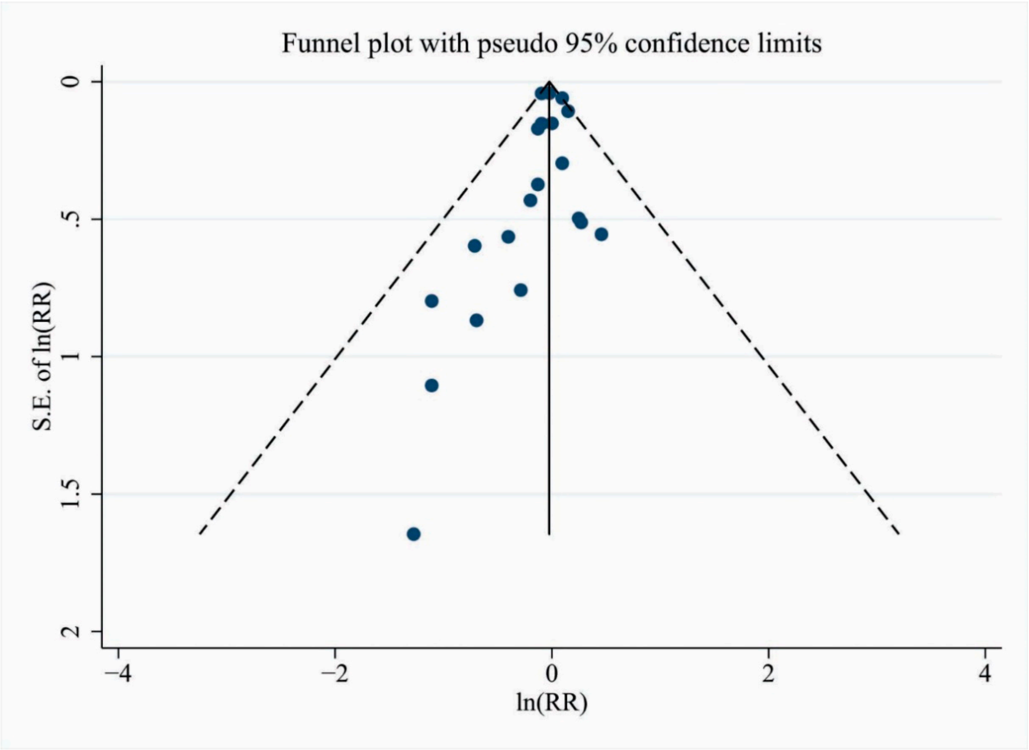

Supplement: Supplementary file 1 [file viruses-15-00765-s001.zip › viruses-2292387-supplementary.pdf]
